# Generalizability and reproducibility of functional connectivity in autism

**DOI:** 10.1186/s13229-019-0273-5

**Published:** 2019-06-24

**Authors:** Jace B. King, Molly B. D. Prigge, Carolyn K. King, Jubel Morgan, Fiona Weathersby, J. Chancellor Fox, Douglas C. Dean, Abigail Freeman, Joaquin Alfonso M. Villaruz, Karen L. Kane, Erin D. Bigler, Andrew L. Alexander, Nicholas Lange, Brandon Zielinski, Janet E. Lainhart, Jeffrey S. Anderson

**Affiliations:** 1 Department of Radiology and Imaging Sciences, University of Utah, Salt Lake City, UT 84108 USA; 2 Interdepartmental Program in Neuroscience, University of Utah, Salt Lake City, UT 84112 USA; 3 Department of Pediatrics, University of Utah, Salt Lake City, UT 84108 USA; 4 Waisman Center, University of Wisconsin-Madison, Madison, WI 53705 USA; 5 Department of Biomedical Engineering, University of Utah, Salt Lake City, UT 84112 USA; 6 Department of Psychiatry, University of Wisconsin-Madison, Madison, WI 53719 USA; 7 Psychology Department and Neuroscience Center, Brigham Young University, Provo, UT 84604 USA; 8 Department of Medical Physics, University of Wisconsin-Madison, Madison, WI 53705 USA; 9 McLean Hospital and Department of Psychiatry, Harvard University, Cambridge, MA 02478 USA; 10 Department of Neurology, University of Utah, Salt Lake City, UT 84132 USA

**Keywords:** Autism spectrum conditions, Resting-state, fMRI, Functional connectivity MRI, Replicability, Reproducibility

## Abstract

**Background:**

Autism is hypothesized to represent a disorder of brain connectivity, yet patterns of atypical functional connectivity show marked heterogeneity across individuals.

**Methods:**

We used a large multi-site dataset comprised of a heterogeneous population of individuals with autism and typically developing individuals to compare a number of resting-state functional connectivity features of autism. These features were also tested in a single site sample that utilized a high-temporal resolution, long-duration resting-state acquisition technique.

**Results:**

No one method of analysis provided reproducible results across research sites, combined samples, and the high-resolution dataset. Distinct categories of functional connectivity features that differed in autism such as homotopic, default network, salience network, long-range connections, and corticostriatal connectivity, did not align with differences in clinical and behavioral traits in individuals with autism. One method, lag-based functional connectivity, was not correlated to other methods in describing patterns of resting-state functional connectivity and their relationship to autism traits.

**Conclusion:**

Overall, functional connectivity features predictive of autism demonstrated limited generalizability across sites, with consistent results only for large samples. Different types of functional connectivity features do not consistently predict different symptoms of autism. Rather, specific features that predict autism symptoms are distributed across feature types.

**Electronic supplementary material:**

The online version of this article (10.1186/s13229-019-0273-5) contains supplementary material, which is available to authorized users.

## Introduction

Atypical resting-state functional connectivity has been proposed as a metric for the pathophysiology of autism [1, 2]. While abnormal functional connectivity has been well-documented in the literature in autism, there are inconsistencies across studies with respect to the spatial distribution of connectivity atypicalities in the brain and even whether abnormal connectivity is too high or too low. There is no consensus on what types of brain connections are abnormal beyond idiosyncratic or atypical connectivity compared to typical development, or how features may evolve with age. Thus, researchers have developed a number of complementary resting-state fMRI analysis methods resulting in multiple approaches to quantifying functional connectivity in autism.

Many of these approaches describe patterns of under- or over-connectivity, in autism compared to controls, between multiple brain regions or networks including corticostriatal [3–8], thalamocortical regions [4, 9], and default mode and salience networks [3, 10–17]. Long-range underconnectivity and short-range overconnectivity have both been hypothesized as a brain abnormality in autism [1, 18–20]. Other models suggest abnormal segregation and integration of resting-state networks [21–25] and idiosyncrasy of connectivity [26, 27]. Aberrant homotopic connectivity in autism compared to controls, measured by interhemispheric correlations, has also been replicated in the literature [28, 29].

Despite this evolution of investigative methods, there are a number of limitations. These approaches are usually examined in isolation, limiting information about overlap or divergence of the information obtained from different methods. Due to conflicting results, there is no accepted role in the clinical practice of brain imaging to constrain diagnosis, prognosis, or therapeutic choices. Converging resting-state functional connectivity evidence is also complicated by the variability of study acquisition parameters, sample selection, preprocessing methods, and analysis methods. As a result, both the extent to which different approaches to functional connectivity in autism are found in similar cohorts of autism participants and whether they correspond to different phenotypic patterns of autistic symptoms remains unclear.

Fortunately, in tandem with advances in resting-state analysis methods comes the growth of publicly available datasets allowing for data collected from many sources to be processed using a uniform analysis pipeline. The Autism Brain Imaging Data Exchange (ABIDE) is one such publicly available dataset that now provides structural and functional brain imaging data collected from more than 25 brain imaging laboratories across the globe [29, 30]. With the availability of multi-site resting-state data comes the ability to test multiple resting-state functional connectivity theories related to autism brain function. This study attempts to distill published differences between individuals with autism and control individuals across a large sample, that includes multiple age ranges and varying cohort characteristics, using proposed methods for resting-state functional magnetic resonance imaging (fMRI) analysis. We do this by addressing the following questions:

1) Which functional connectivity features are reproducible across a large multi-site sample of participants with autism?

2) Can significant features of autism from a large sample that are heterogeneous across sites be identified within a single sample using high-temporal resolution, long-duration modern acquisition techniques?

3) To what extent do distinct functional connectivity features track together in the same participants, or do different features represent different aspects or endophenotypes of autism?

We present these findings across individual research sites included in the ABIDE dataset, the combined ABIDE I data release, the combined ABIDE II data release, and the full ABIDE data sample. Our goal is to compare and quantify the generalizability and reproducibility of functional connectivity methods in individuals with autism and identify the extent to which different methodological approaches identify complementary information.

## Methods

### Participants

The ABIDE dataset included an initial (ABIDE I) and a second data release (ABIDE II). A total of 17 sites contributed 1112 individuals in the ABIDE I release including 539 individuals with autism and 573 controls. Data from ABIDE II was compiled across 19 sites with a total of 1114 individual datasets comprised of 521 individuals with autism and 593 controls. For site-specific details on both diagnostic criteria and more detailed characterization of clinical and behavioral phenotypes, see [31]. Both ABIDE I and ABIDE II contain data supplied by the University of Utah. The current study also includes a replication sample (Utah cohort) that consisted of 52 males with autism and 38 control males with high-temporal resolution and long duration resting-state scan data acquired as part of a larger longitudinal study of autism aimed at investigating brain development across the adolescent and adult lifespan.

### Data processing

#### Abide

Structural data from the ABIDE dataset were processed using FreeSurfer (v6.0.0). A detailed description of the FreeSurfer pipeline can be found on the FreeSurfer website [32]. Preprocessing of the ABIDE fMRI blood-oxygen-level-dependent (BOLD) data was performed in MATLAB (MathWorks, Natick, MA, USA) using SPM12 (Wellcome Department of Imaging Neuroscience, London, UK). All images were corrected for motion using a realign and unwarp procedure. Each participant’s BOLD images were coregistered to their individual MPRAGE anatomic image sequence. Phase-shifted soft tissue correction (PSTCor) [28] was used to regress participant motion parameters, eroded white matter, eroded cerebral spinal fluid, and soft tissues of the face and calvarium. Eroded masks were obtained by removing all voxels from white matter and CSF masks that were adjacent to a voxel not in the mask. Volume censoring (scrubbing) was performed with removal of volumes before and after mean framewise displacement head motion greater than 0.3 mm [33]. Only participants with ≥ 50% volumes remaining after scrubbing were considered for further analysis. In the ABIDE I data release, 1112 participants were analyzed and 419 were removed (220 due to motion, 199 due to other quality issues). In the ABIDE II data release, 1114 participants were analyzed and 405 were removed (217 due to motion, 188 due to other quality issues). Individual research sites with less than 10 participants remaining after initial quality control were removed from further analysis. During volume censoring, 14 ± 1% of volumes were censored for neurotypical subjects and 18 ± 1% of volumes were censored for autism subjects across sites (*t* (1400) = − 5.12, *p* < .001, 95% CI [−.05–.02]). Following preprocessing, quality control was completed by imaging experts that consisted of visual inspection of each participant’s neuroimaging data for successful completion of the preprocessing pipeline and image quality. Specifically, each image was checked for brain coverage and any scanner artifacts such as ghosting and indications of high movement. This was completed by viewing representative images on each plane. Images with obvious quality issues were immediately rejected. Images with suspected quality issues were subjected to further review requiring the reviewer to scroll through each volume in the image. A second instance of quality control consisted of verifying normalization by overlaying T1 images on processed BOLD data. Finally, the FreeSurfer subcortical segmentation was overlaid on representative images and assessed for quality. No edits were made to subcortical segmentations. Data not meeting visual inspection was removed from further analysis.

#### Utah cohort

Detailed information regarding the acquisition and processing of this cohort can be found in King et al. [34]. Briefly, resting-state functional images were acquired using a multi-band multi-echo echo-planar sequence (TR = 1553 milliseconds; flip angle = 65°; inplane acceleration factor = 2; fields of view = 208 mm; 72 axial slices; resolution = 2.0 mm isotropic; multi-band acceleration factor = 4; partial Fourier = 6/8; bandwidth = 1850 Hz; 3 echoes with TEs of 12.4 ms, 34.28 ms, and 56.16 ms; and effective TE spacing = 22 ms) with two acquisitions of 590 images (15 min, 27 s) each (one left-to-right and one right-to-left), each with 3 volumes representing different echo times. Structural images consisted of an MP2RAGE sequence with isotropic 1 mm resolution (TR = 5000 ms, TE = 2.91 ms). Structural data were processed using FreeSurfer (v6.0.0) using the default processing pipeline with the input image derived by multiplying the MP2RAGE uniform image by the proton-density-weighted image [35]. Visual inspection of the finished product was completed to ensure subcortical segmentation quality. No subjects included in this cohort required any alterations to segmentation. Analysis of resting-state data was conducted using a multi-echo independent component analysis (ME-ICA) pipeline included in the Analysis of Functional NeuroImages (AFNI) open-source environment [36, 37].

#### Human connectome project

In order to establish normalized measures of resting-state functional connectivity patterns following typical development, FIX ICA processed fcMRI data for 1003 participants and diffusion tensor imaging (DTI) data from 1021 participants from the Human Connectome Project (ages 22–35) [38] were included in this analysis. As data in the ABIDE dataset includes a variety of acquisition parameters and data quality, some not yielding a very high resolution, relying on normative data created from dissimilar resting-state acquisitions was not ideal. Due to the high quality of the Human Connectome Project dataset, it was selected in order to model a representative typically developing the brain in a number of functional connectivity measures. Similarly, the DTI data included in the Human Connectome Project dataset provide high-quality data from which structural path lengths can be estimated. Regrettably, the ABIDE dataset does not include DTI data at this time.

#### Resting-state fMRI regions of interest

Parcellation of the brain regions of interest (ROI) from which functional connectivity values were derived was conducted as previously described [34, 39]. This process was completed for all three datasets. Briefly, for each participant, time series data were extracted from 333 cortical regions [40], 14 participant-specific subcortical regions from FreeSurfer-derived segmentation [41] (bilateral thalamus, caudate, putamen, amygdala, hippocampus, pallidum, and nucleus accumbens), and 14 bilateral cerebellar representations of a 7-network parcellation [42] with each network treated as a single region of interest. When combined, this parcellation scheme incorporates major cortical, subcortical, and cerebellar gray matter ROIs numbering 361 regions in total [39, 43].

#### Resting-state data analysis

Between-group analyses were conducted for each research site within the ABIDE dataset, for participants included in the ABIDE I (A1) and ABIDE II (A2) data releases, as well as the full ABIDE sample (ABIDE). All analyses were then duplicated in the Utah replication cohort.

General linear models were used to compare group differences in functional connectivity while controlling for age and mean head motion (both cohorts) as well as sex and site when applicable (ABIDE). Correction for multiple comparisons was completed using the false discovery rate (*q* (FDR) < .05). Unless stated otherwise, FDR corrections presented in figures were conducted on the combined *p* values across sites, A1, A2, ABIDE, and the Utah cohort. Pearson correlations were used to test for associations between neuroimaging findings and both behavioral and cognitive factors.

BrainNet Viewer was used to create brain images included in figures [44]. For display purposes, a smoothed gray matter mask was used, and each gray matter voxel was assigned to one of the 333 regions based on nearest proximity. Statistical analyses were performed in the MATLAB computing environment (MathWorks, Natick, MA, USA) and SPSS software (version 25) for Mac OS X.

### Resting-state functional connectivity analysis methods

A total of eight resting-state functional connectivity analysis methods were selected to compare connectivity differences between individuals with autism and controls. Methods were selected based on an extensive review of the literature.

#### Positive versus negative functional connectivity

Specific differences in task fMRI have been identified in brain regions responsible for control of inhibition [45], and specific abnormalities have been observed in autism for negatively correlated (anticorrelated) connections [18] and in negative BOLD responses in networks [46]. In order to establish normalized connectivity strength values for each of the 361 Gy matter region pairs from an independent sample of young adults, functional connectivity MRI values from the Human Connectome Project dataset were averaged across participants. These values were then grouped into 40 bins of .02 ranging between −.2 and .6. The four most negative bins contained no data and were removed. Mean functional connectivity values for each participant in the ABIDE dataset were extracted across the same 361 ROI pairs and average values for the connections in each bin were computed for each participant. These values were then compared between individuals with autism and controls in the ABIDE and Utah cohorts for each bin.

#### Short- and long-range functional connectivity

Between-group differences in short- and long-range functional connectivity in the ABIDE sample were calculated using two methods. First, normalized path lengths previously established using DTI data from the Human Connectome Project dataset were calculated between each pair of ROIs for which a structural connection was identified. A group average DTI template was constructed from 1021 participants using q-space diffeomorphic reconstruction with deterministic fiber tracking algorithm with 50,000 whole brain seeding in DSI studio [47, 48]. Data from 47,903 fibers were intersected with the 361-region atlas previously described to obtain structurally connected regions. Connections were binned into 10 bins of 50 mm ranging from 0 to 500 mm. Functional connectivity region pair values from the ABIDE dataset were then compared between individuals with autism and controls for each of the path length bins for region pairs identified from the Human Connectome Project dataset.

Second, this process was repeated using the Euclidian distance between ROI centroids in the Human Connectome Project dataset for all ROI pairs. Bins were created representing these distances ranging between 0 and 165 mm for every 5 mm. The first bin was empty resulting in a total of 32 bins. Values from the ABIDE dataset were then compared between individuals with autism and controls for each of the Euclidian distance path length bins for region pairs identified from the Human Connectome Project dataset. This process was replicated in the Utah cohort.

#### Homotopic connectivity

Region of interest centroids were used to identify homotopic region pairs by first inverting the x coordinate from each of the 361 ROI centroids and determining which of the non-inverted ROIs represented the ROI with the minimum distance. The same homotopic region pairs were used for all the datasets in the analysis.

#### Corticostriatal connectivity

Ipsilateral corticostriatal connectivity was evaluated by extracting functional connectivity values between participant-specific, FreeSurfer-derived left and right caudate, putamen, globus pallidus, and nucleus accumbens subcortical gray matter ROIs to all intrahemispheric cortical ROIs. Cortical ROIs numbered 161 in the left hemisphere and 172 in the right hemisphere. Between-group differences in corticostriatal connectivity were then explored for each of the eight subcortical gray matter ROIs for both the ABIDE and Utah cohorts.

#### Thalamocortical connectivity

Thalamocortical connectivity was evaluated by extracting functional connectivity values between left and right thalamus to all ipsilateral cortical ROIs as described above. Between-group differences in thalamocortical connectivity were then explored for both the ABIDE and Utah cohorts.

#### Idiosyncrasy

In order to determine the idiosyncrasy of each participant for both the ABIDE and Utah cohorts, an averaged idiosyncrasy value (*s*^2^) was assigned to each participant by calculating the variance for each participant’s functional connectivity values for each of the 361 region pairs (*x*_*i*_) and using the averaged Human Connectome Project data for the 361 region pairs as the reference mean ($\overline{x}\Big)$ and the total number of elements (361 × 361) as *n* in the variance equation.

$$ {s}^2=\frac{\sum {\left({x}_i-\overline{x}\right)}^2}{n-1} $$

These variance values were then compared between individuals with autism and controls.

#### Abnormal segregation/integration

Network modularity was used to assess segregation while integration was assessed utilizing global efficiency. In order to compute modularity for each participant in the ABIDE and Utah cohorts, time series data were first normalized to fall between −1 and 1. Next, the MATLAB script modularity_und.m, from the Brain Connectivity Toolbox, was used to modularize the data [49]. Each participant’s maximized modularity value was stored for between-group analyses. Participant-specific global efficiency was then computed on inverted normalized values from the previous step using the Brain Connectivity Toolbox’s efficiency_wei.m script. Modularity and global efficiency were then compared between individuals with autism and controls in the ABIDE and Utah cohorts.

#### Default mode and salience network connectivity

For each participant in the ABIDE and Utah cohorts, functional connectivity values from the 333 ROI Gordon et al. [40] parcellation that had been previously assigned to the default mode (41 cortical ROIs) and salience (36 total cortical ROIs including 4 from the salience network and 32 from the dorsal attention network) networks in the referenced study were evaluated for within and between network connectivity.

#### Lagged connectivity

Recent reports have suggested that alterations in lagged functional connectivity may be abnormal in autism [34, 50]. A closely related finding that the shape and parameters of the hemodynamic response function may be altered in autism [51, 52], similar to alterations in the HRF in stroke [53], may be related to both neural and non-neural factors. A study of the width of the autocorrelation function in resting state fMRI, determined by lagged connectivity, found a close mutual relationship between parameters of the HRF, autocorrelation width, cognitive processing speed, and reaction times on functional tasks [39]. A neural contribution to lagged connectivity in autism may relate to prolonged neural activity in autism and could be associated with recent reports of brain hyperstability in autism [54–57].

Based on findings detailed in King et al. [34] that found between-group differences in a measure of lag-based functional connectivity, these lag-based findings were also included in feature selection in order to compare method similarities. Lag-based connectivity values considered for feature selection from King et al. [34] represented functional connectivity values at 6 (ABIDE) or 6.212 (Utah cohort) second lags. Refer to King et al. [34] for details related to processing. Briefly, cross-correlation curves were computed using the repetition time (TR) from each individual research site, with cubic spline interpolation to identify correlation at zero lag and positive and negative lags (seconds).

#### Feature selection

In order to further determine which of the methods provide analogous findings in individuals with autism compared to controls, a number of relevant features were selected for further analysis. Feature selection consisted of identifying functional connectivity features that demonstrated a significance value of *p* < .05 (uncorrected) for each of the examined methods. Methods used to evaluate idiosyncrasy and abnormal segregation and integration provided only one *p* value for each participant and were not included in feature selection.

Feature selection was conducted on *p* values in the combined ABIDE sample. Region pairs identified using feature selection were also evaluated in the Utah cohort. A linear regression model was employed to regress out effects of site, age, sex, and mean head motion (root-mean-square) in the ABIDE sample, and age and mean head motion in the Utah cohort. As the proportion of censored volumes was significantly different between individuals with autism and controls, a post hoc analysis was also completed using percent motion-free volumes as a regressor in place of mean head motion. In order to compare similarities in findings related to methods, Pearson correlations were calculated between all features as well as features averaged across methods in individuals with autism from the combined ABIDE sample. In the combined ABIDE autism sample, Pearson correlations (unthresholded) were also calculated between available reported performance, verbal, and full-scale intelligence quotient (IQ) scores (see http://fcon_1000.projects.nitrc.org/indi/abide/ for details related to IQ measures), Autism Diagnostic Observation Schedule (ADOS) severity, total and subscale scores (Gotham score from ABIDE I; ADOS-2 scores from ABIDE II) [58, 59], Social Responsiveness Scale (SRS) raw total and subscale scores [60], and Autism Diagnostic Interview-Revised (ADI) total, subscale, and onset scores [61].

## Results

### Resting-state functional connectivity

**Fig. 1:**
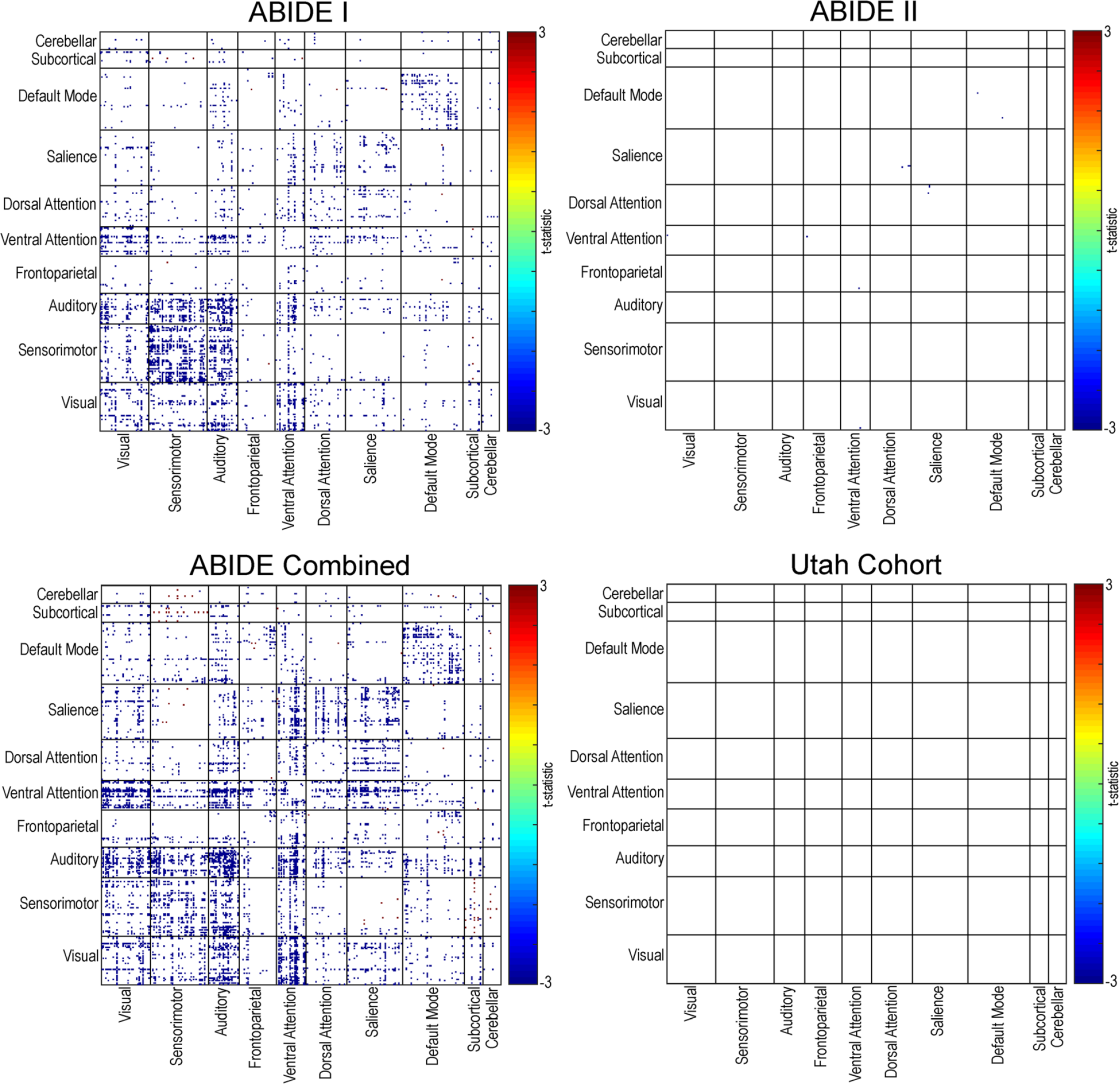
ABIDE resting-state functional connectivity. Distribution of between-group resting-state findings for a 361 region of interest parcellation in ABIDE I, ABIDE II, a combined ABIDE dataset, and a high-temporal resolution replication sample (Utah cohort). Between-group differences were calculated using a general linear model controlling for age, sex, mean head motion, and site in the ABIDE dataset, and age and mean head motion in the Utah cohort (*q* (FDR) < .05). Cooler colors represent autism < controls

A total of 1402 participants from the combined ABIDE dataset, that included 693 participants across 11 sites from the ABIDE I sample, and 709 participants across 14 sites from the ABIDE II sample were analyzed. An additional replication sample of 90 participants from a separate Utah cohort is also included (see Table 1 and Additional file 1: Tables S1–S4). Widespread decreases in functional connectivity (*q* (FDR) < .05) were found in individuals with autism compared to controls in the ABIDE I and combined ABIDE samples, whereas few significant region pairs were found in the ABIDE II sample. No multiple comparison corrected significant findings were found in the Utah cohort (see Fig. 1). Site-specific analyses of resting-state functional connectivity revealed extensive between-site variability in the directionality of results (autism > control; autism < control; *p* < .05, uncorrected) (see Additional file 1: Figures S1–S7). Only one site (ONRC) demonstrated multiple comparison corrected findings (*q* (FDR) < .05) which were located primarily in default mode and frontoparietal network connections.

**Table 1:** Demographic information for the ABIDE dataset and the Utah cohort

	*N* =			Male % (total sample)			Age			Motion		
	ASD^a^	TD^b^	Total	ASD	TD	Total	ASD	TD	*p*	ASD	TD	*p*
ABIDE I	295	398	693	37.5	46.6	84.1	16.7 ± 7.3	16.5 ± 6.6	.696	.11 ± .05	.09 ± .05	<.001
ABIDE II	284	425	709	33.7	40.8	74.5	13.4 ± 5.9	13.7 ± 6.7	.495	.11 ± .06	.10 ± .05	.003
ABIDE	579	823	1402	35.6	43.7	79.2	15.1 ± 6.9	15.1 ± 6.8	.963	.11 ± .06	.10 ± .05	<.001
Utah	52	38	90	100	100	100	27.7 ± 8.7	27.1 ± 7.5	.716	.10 ± .04	.07 ± .02	.002

^a^Autism Spectrum Disorder

^b^Typically Developing

### Comparison of resting-state functional connectivity methods

No one method demonstrated wholly reproducible findings across sites and datasets when controlling for age, sex, mean head motion, and site.

#### Positive versus negative functional connectivity

**Fig. 2:**
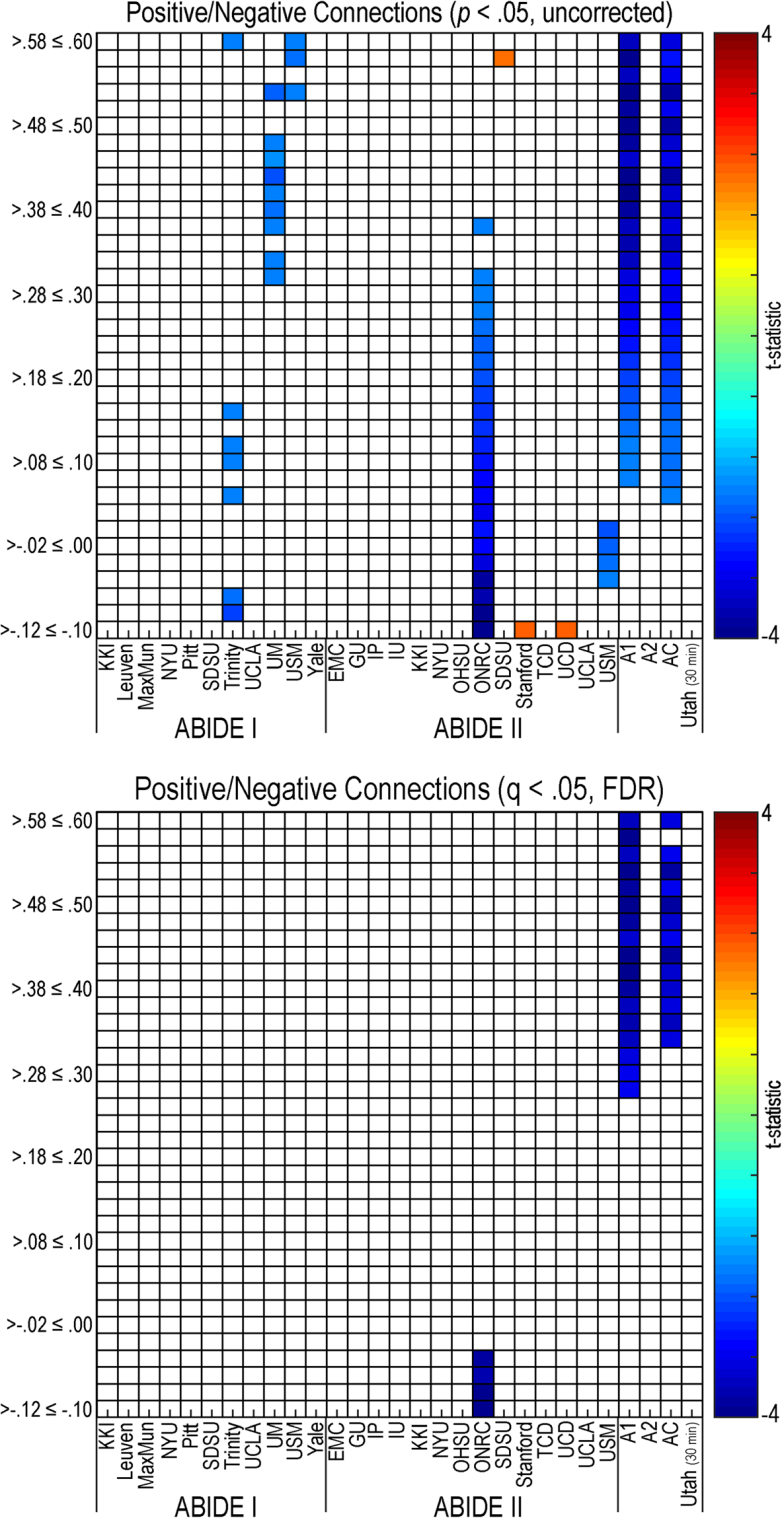
Positive/negative resting-state functional connectivity in autism. Positive and negative functional connectivity values from a 361 region of interest parcellation were placed into bins (*y*-axis) for all sites with the ABIDE dataset, ABIDE I, ABIDE II, a combined ABIDE dataset, and a high-temporal resolution replication sample (Utah cohort). Between-group differences were calculated using a general linear model controlling for age, sex, mean head motion, and site in the ABIDE datasets, and age and mean head motion in the site-specific and Utah cohorts. Uncorrected results are reported in the top panel (*p* < .05). Multiple comparison corrected findings are reported in the lower panel (*q* (FDR) < .05). Cooler colors represent autism < controls

Positive and negative functional connectivity demonstrated primarily decreased connectivity in individuals with autism compared to controls (see Fig. 2). This pattern was found in both positive and negative connectivity in the Trinity (A1), ONRC (A2), and USM (A2) research sites and positive connectivity only in UM (A1), USM (A2), ABIDE I, and the combined ABIDE dataset (*p* < .05, uncorrected). Significant negative connectivity findings (autism < controls) meeting multiple comparison correction (*q* (FDR) < .05) were found only in one site, ONRC (A2), while the ABIDE I and combined ABIDE datasets demonstrated significant positive connectivity. No multiple comparison corrected significant findings were found in the ABIDE II dataset.

#### Short- and long-range connectivity

**Fig. 3:**
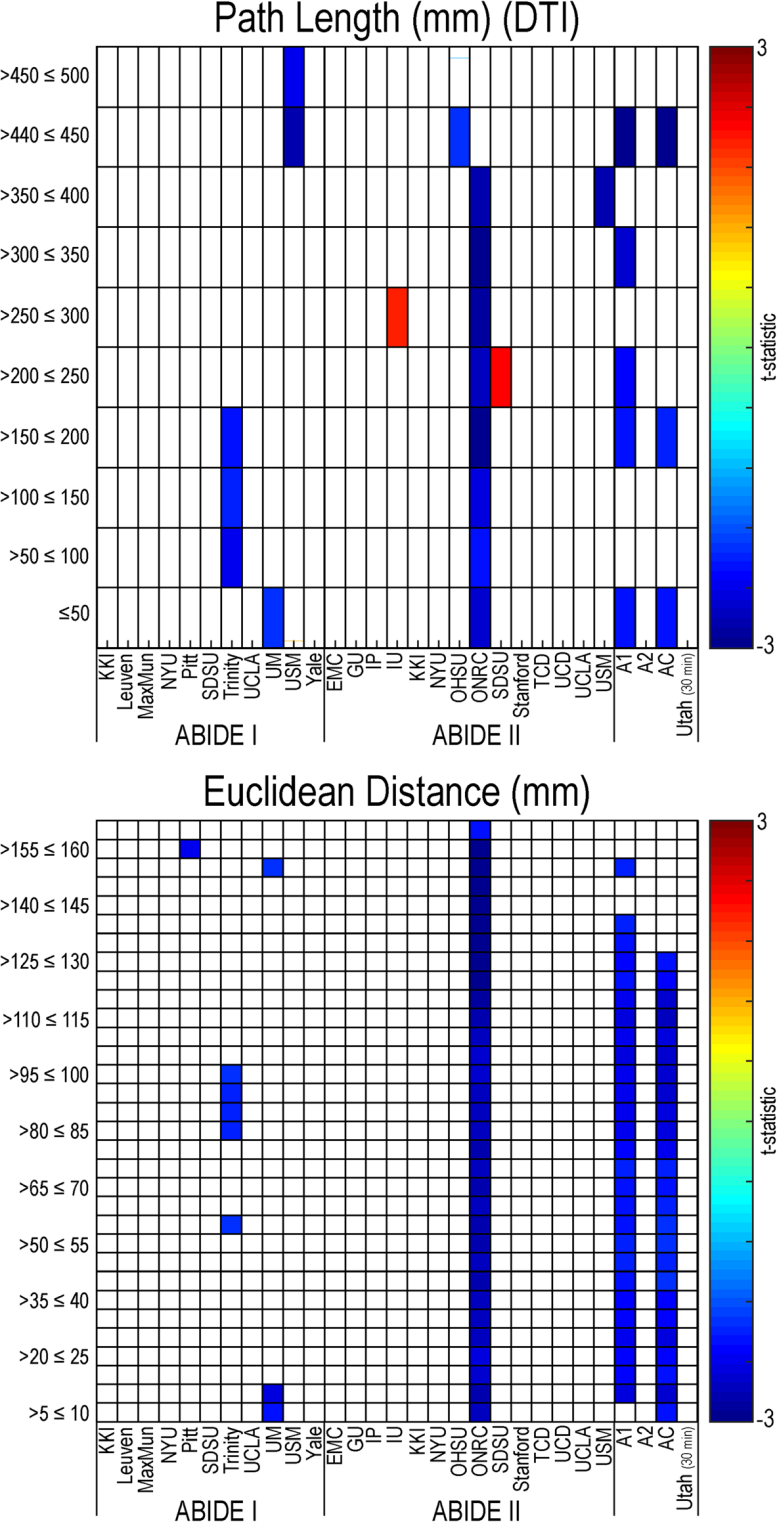
Short- and long-range resting-state functional connectivity in autism. Distance values calculated for a 361 region of interest parcellation were placed into bins (*y*-axis), using DTI white matter path lengths (top panel) and Euclidean distance (bottom panel), for all sites within the ABIDE dataset, ABIDE I, ABIDE II, a combined ABIDE dataset, and a high-temporal resolution replication sample (Utah cohort). Between-group differences were calculated using a general linear model controlling for age, sex, mean head motion, and site in the ABIDE datasets, and age and mean head motion in the site-specific and Utah cohorts. (*p* < .05, uncorrected). Cooler colors represent autism < controls

Based on average path lengths established using DTI white matter tracts from the Human Connectome Project dataset, both short- and long-range functional connectivity were found to be primarily decreased in individuals with autism compared to controls; however, none of these findings met multiple comparison correction (see Fig. 3). Euclidean distance between gray matter ROIs was also established based on averages from the Human Connectome Project dataset. Again, both short- and long-range connectivity were decreased in individuals with autism compared to controls. No findings based in Euclidean distance passed multiple comparison correction.

#### Homotopic connectivity

**Fig. 4:**
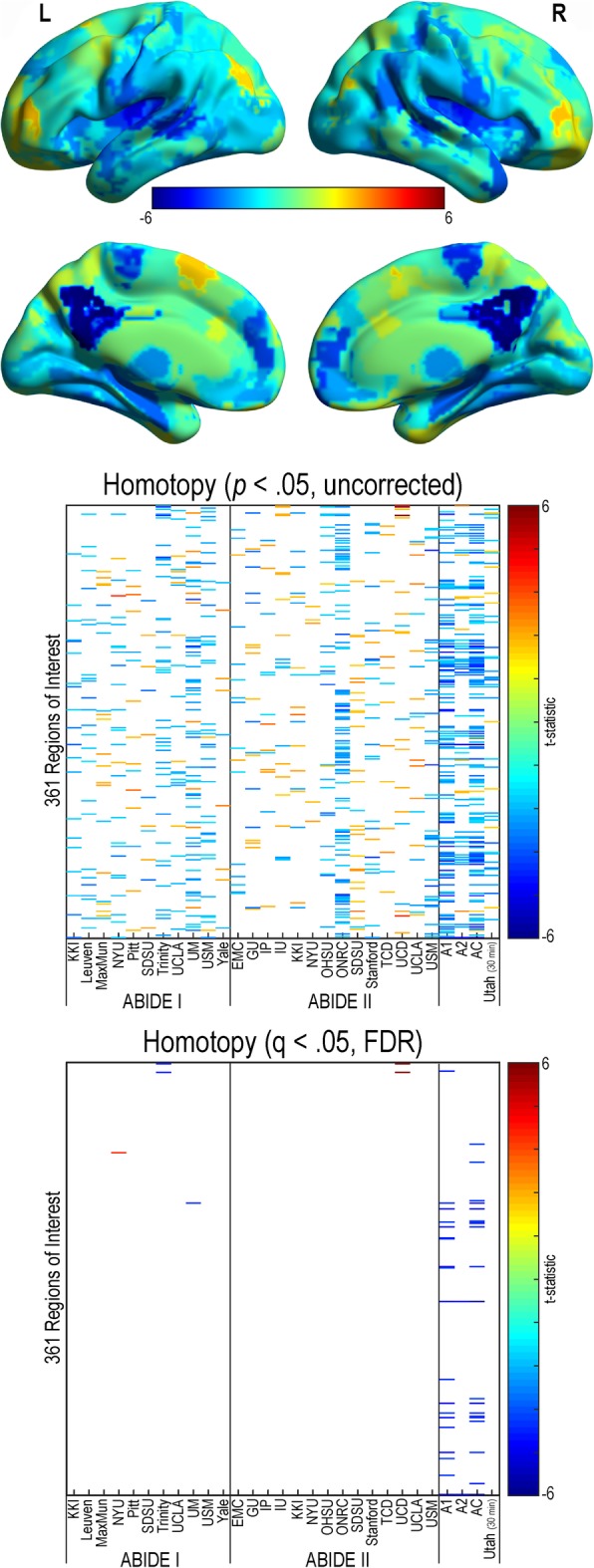
Homotopic resting-state functional connectivity in autism. Homotopic values calculated for a 361 region of interest parcellation for all sites within the ABIDE dataset, ABIDE I, ABIDE II, a combined ABIDE dataset, and a high-temporal resolution replication sample (Utah cohort). Between-group differences were calculated using a general linear model controlling for age, sex, mean head motion, and site in the ABIDE datasets, and age and mean head motion in the site-specific and Utah cohorts. *t* statistic from ABIDE combined sample is shown overlaid on a template brain image (above). Uncorrected results are reported in the middle panel (*p* < .05). Multiple comparison corrected findings are reported in the lower panel (*q* (FDR) < .05). Cooler colors represent autism < controls

Homotopic functional connectivity findings were not generally reproducible across sites (see Fig. 4). The majority of sites demonstrated widespread decreases in homotopic connectivity in individuals with autism compared to controls; however, some sites demonstrated both increased and decreased homotopic connectivity in individuals with autism compared to controls (*p* < .05, uncorrected). When controlling for multiple comparisons (q (FDR) < .05), decreased homotopic connectivity for multiple region pairs was found in the ABIDE I, ABIDE II, and combined ABIDE datasets. Sparse FDR-corrected findings were found in individual research sites.

#### Corticostriatal connectivity

**Fig. 5:**
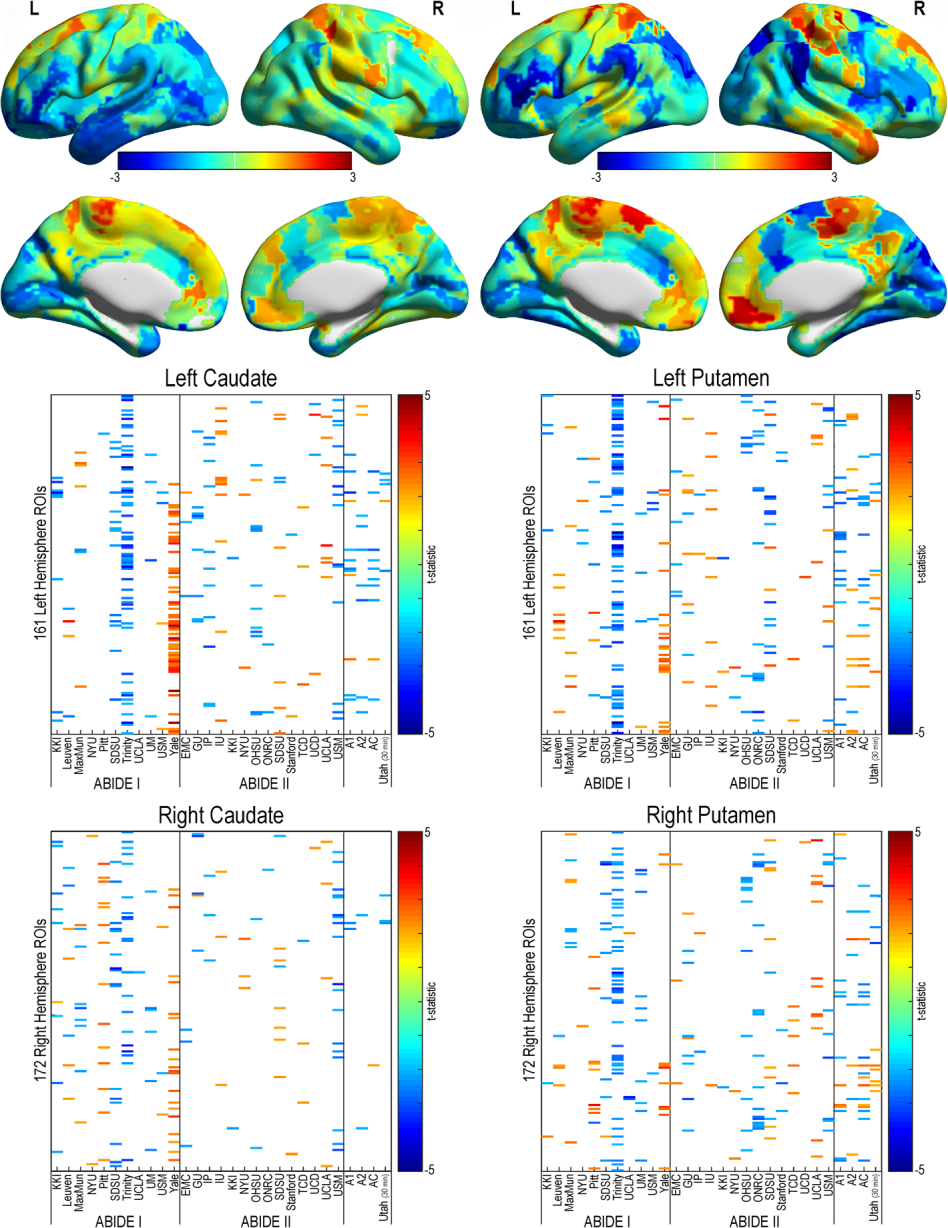
Corticostriatal resting-state functional connectivity in autism (caudate and putamen). Functional connectivity was calculated between participant specific bilateral caudate (left column) and putamen (right column) and ipsilateral cortical regions from a 333 region of interest parcellation for all sites within the ABIDE dataset, ABIDE I, ABIDE II, a combined ABIDE dataset, and a high-temporal resolution replication sample (Utah cohort). Between-group differences were calculated using a general linear model controlling for age, sex, mean head motion, and site in the ABIDE datasets, and age and mean head motion in the site-specific and Utah cohorts (*p* < .05, uncorrected). Cooler colors represent autism < controls. Top panel represents findings overlaid across the cortex (unthresholded *t* statistic)

**Fig. 6:**
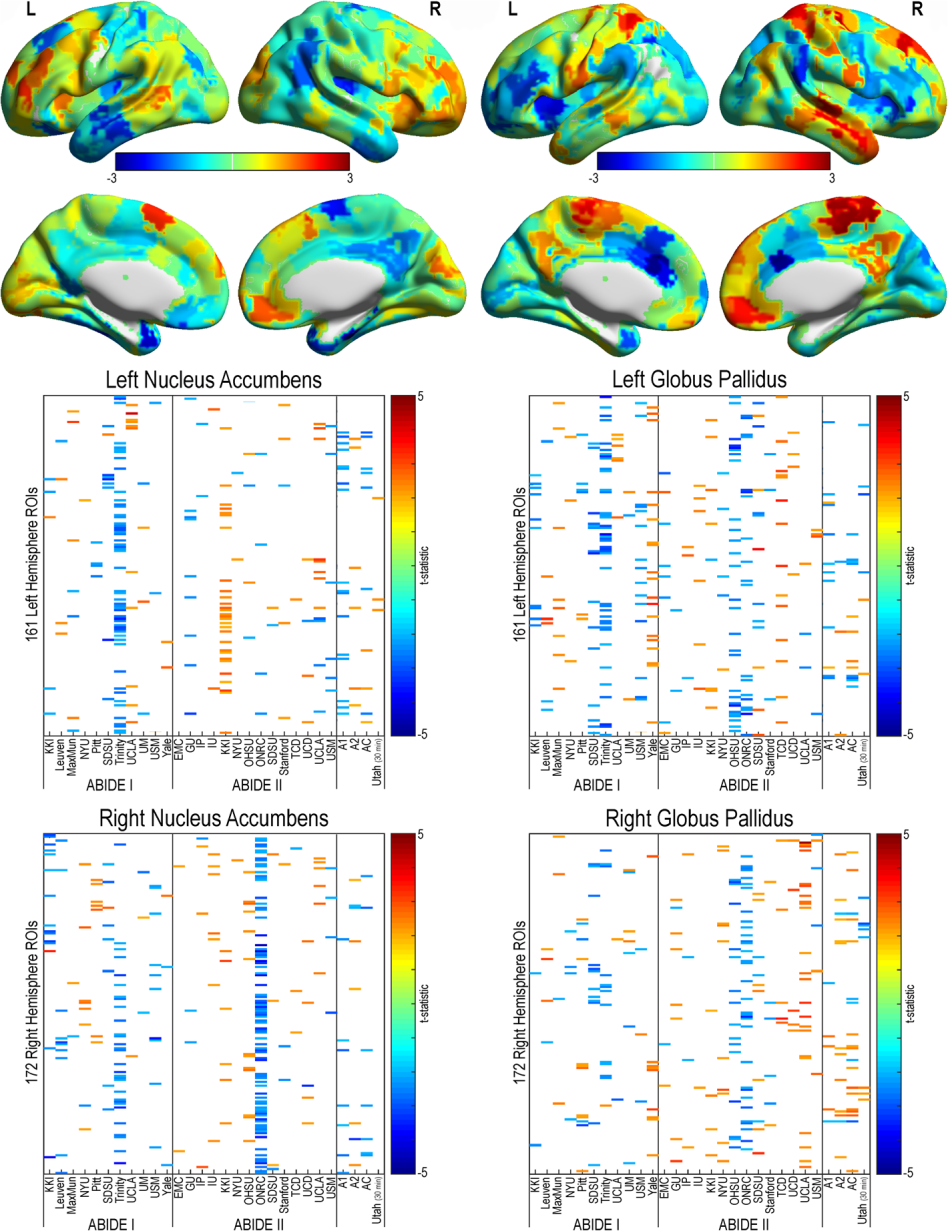
Corticostriatal resting-state functional connectivity in autism (nucleus accumbens and globus pallidus). Functional connectivity was calculated between participant-specific bilateral nucleus accumbens (left column) and globus pallidus (right column) and ipsilateral cortical regions from a 333 region of interest parcellation for all sites within the ABIDE dataset, ABIDE I, ABIDE II, a combined ABIDE dataset, and a high-temporal resolution replication sample (Utah cohort). Between-group differences were calculated using a general linear model controlling for age, sex, mean head motion, and site in the ABIDE datasets, and age and mean head motion in the site-specific and Utah cohorts (*p* < .05, uncorrected). Cooler colors represent autism < controls. Top panel represents findings overlaid across the cortex (unthresholded *t* statistic)

No consistent reproducible patterns were revealed in corticostriatal functional connectivity region pairings (see Figs. 5 and 6). Indeed, both increased and decreased findings were found when comparing individuals with autism to controls (*p* < .05, uncorrected). No findings passed correction for multiple comparisons.

#### Thalamocortical connectivity

**Fig. 7:**
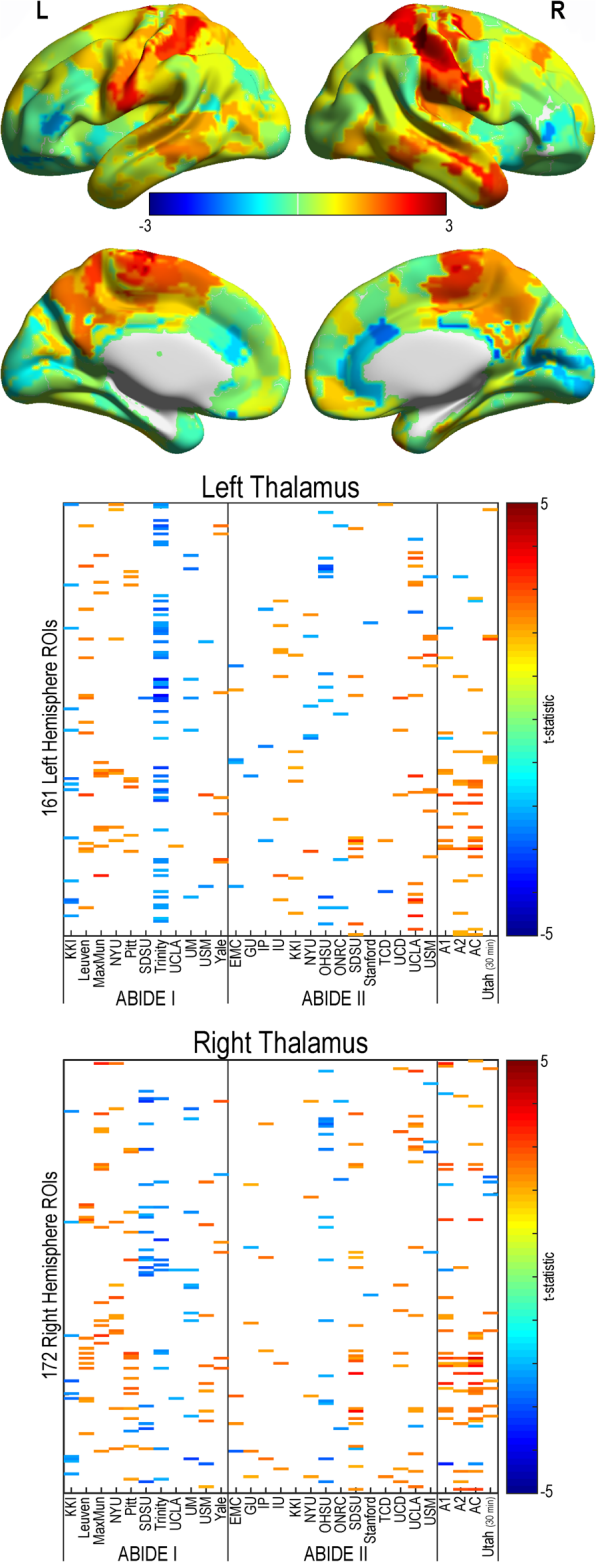
Thalamocortical resting-state functional connectivity in autism. Functional connectivity was calculated between participant-specific bilateral thalamus and ipsilateral cortical regions from a 333 region of interest parcellation for all sites within the ABIDE dataset, ABIDE I, ABIDE II, a combined ABIDE dataset, and a high-temporal resolution replication sample (Utah cohort). Between-group differences were calculated using a general linear model controlling for age, sex, mean head motion, and site in the ABIDE datasets, and age and mean head motion in the site-specific and Utah cohorts (*p* < .05, uncorrected). Cooler colors represent autism < controls. Top panel represents findings overlaid across the cortex (unthresholded *t* statistic)

A pattern of primarily increased connectivity in autism compared to controls was found for the majority of research sites, ABIDE I, ABIDE II, and the combined ABIDE datasets (see Fig. 7). This pattern was also observed in the Utah cohort (*p* < .05, uncorrected). However, no findings passed correction for multiple comparisons.

#### Idiosyncrasy

**Fig. 8:**
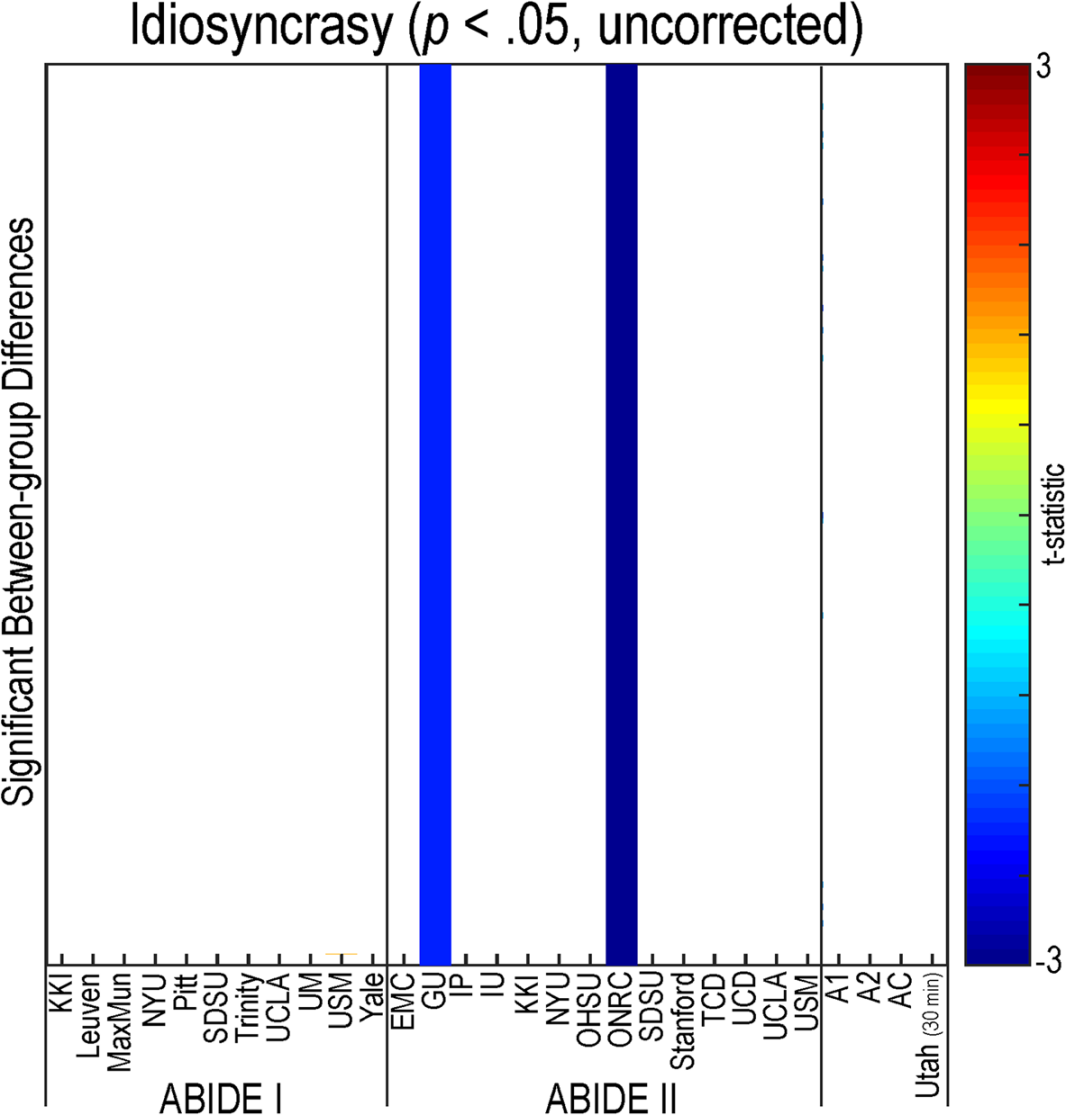
Idiosyncrasy of resting-state functional connectivity in autism. Variance was calculated between each participant’s functional connectivity values for each of the 361 region pairs compared to averaged data from the Human Connectome Project for all sites within the ABIDE dataset, ABIDE I, ABIDE II, a combined ABIDE dataset, and a high-temporal resolution replication sample (Utah cohort). Between-group differences were calculated using a general linear model controlling for age, sex, mean head motion, and site in the ABIDE datasets, and age and mean head motion in the site-specific and Utah cohorts (*p* < .05, uncorrected). Cooler colors represent autism < controls

Normalized functional connectivity values for 361 region pairs were established using the Human Connectome Project dataset (see Fig. 8). Only two research sites (GU (A1) and ONRC (A2)) demonstrated decreased idiosyncrasy in individuals with autism compared to controls when compared to the Human Connectome Project dataset (*p* < .05, uncorrected). No significant findings were observed that passed multiple comparison correction.

#### Abnormal segregation/integration

**Fig. 9:**
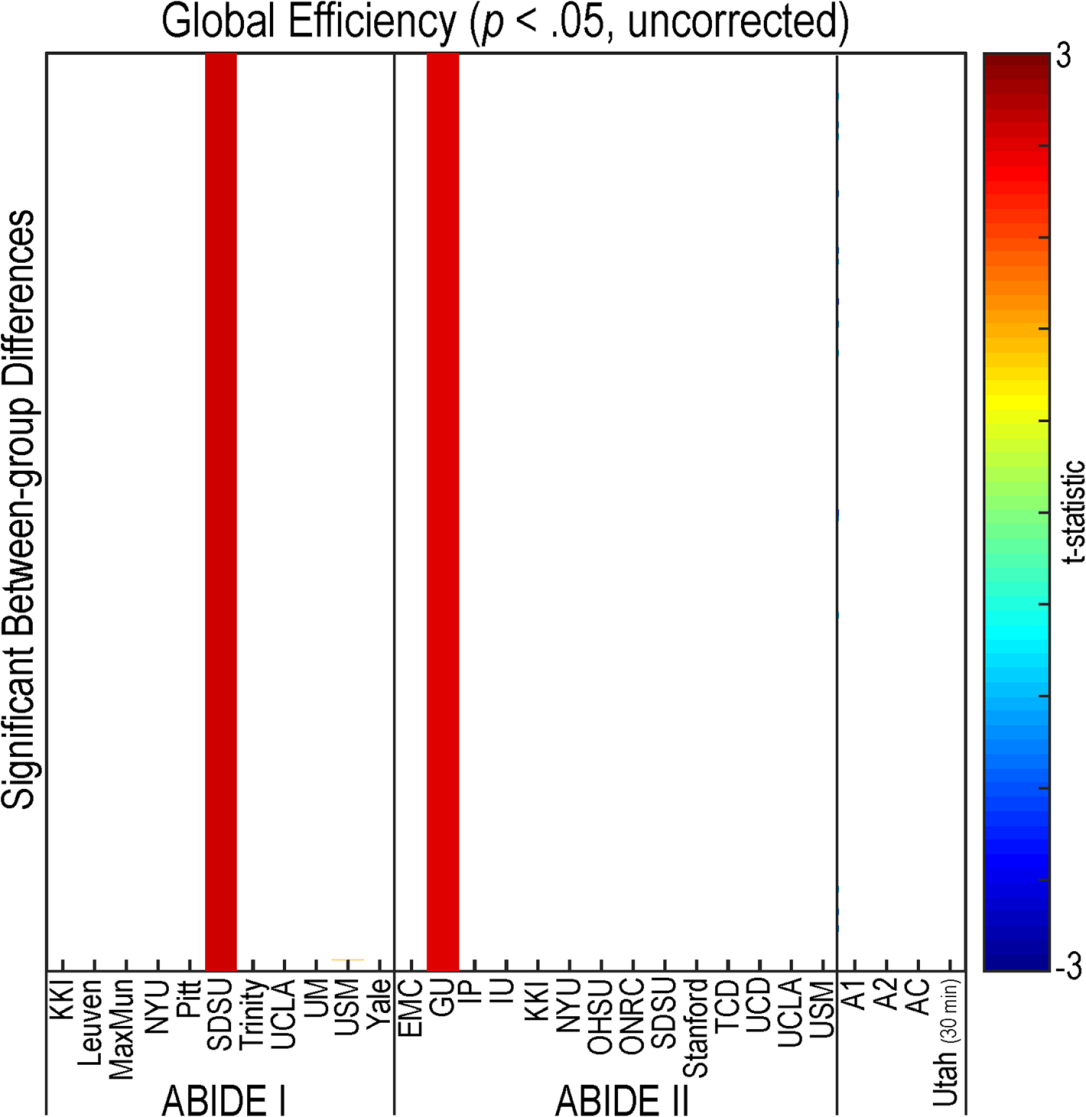
Integration of resting-state functional connectivity in autism. Global efficiency values were calculated as an indicator of integration for each participant in all sites within the ABIDE dataset, ABIDE I, ABIDE II, a combined ABIDE dataset, and a high-temporal resolution replication sample (Utah cohort). Between-group differences were calculated using a general linear model controlling for age, sex, mean head motion, and site in the ABIDE datasets, and age and mean head motion in the site-specific and Utah cohorts (*p* < .05, uncorrected). Cooler colors represent autism < controls

Abnormal segregation analyses, as estimated using a metric of modularity, revealed no findings. Functional integration, as assessed by global efficiency, demonstrated increases in individuals with autism compared to controls in two research sites (SDSU (A1) and GU (A2)) (see Fig. 9). No significant findings were observed for global efficiency that passed multiple comparison correction.

#### Default mode and salience network connectivity

**Fig. 10:**
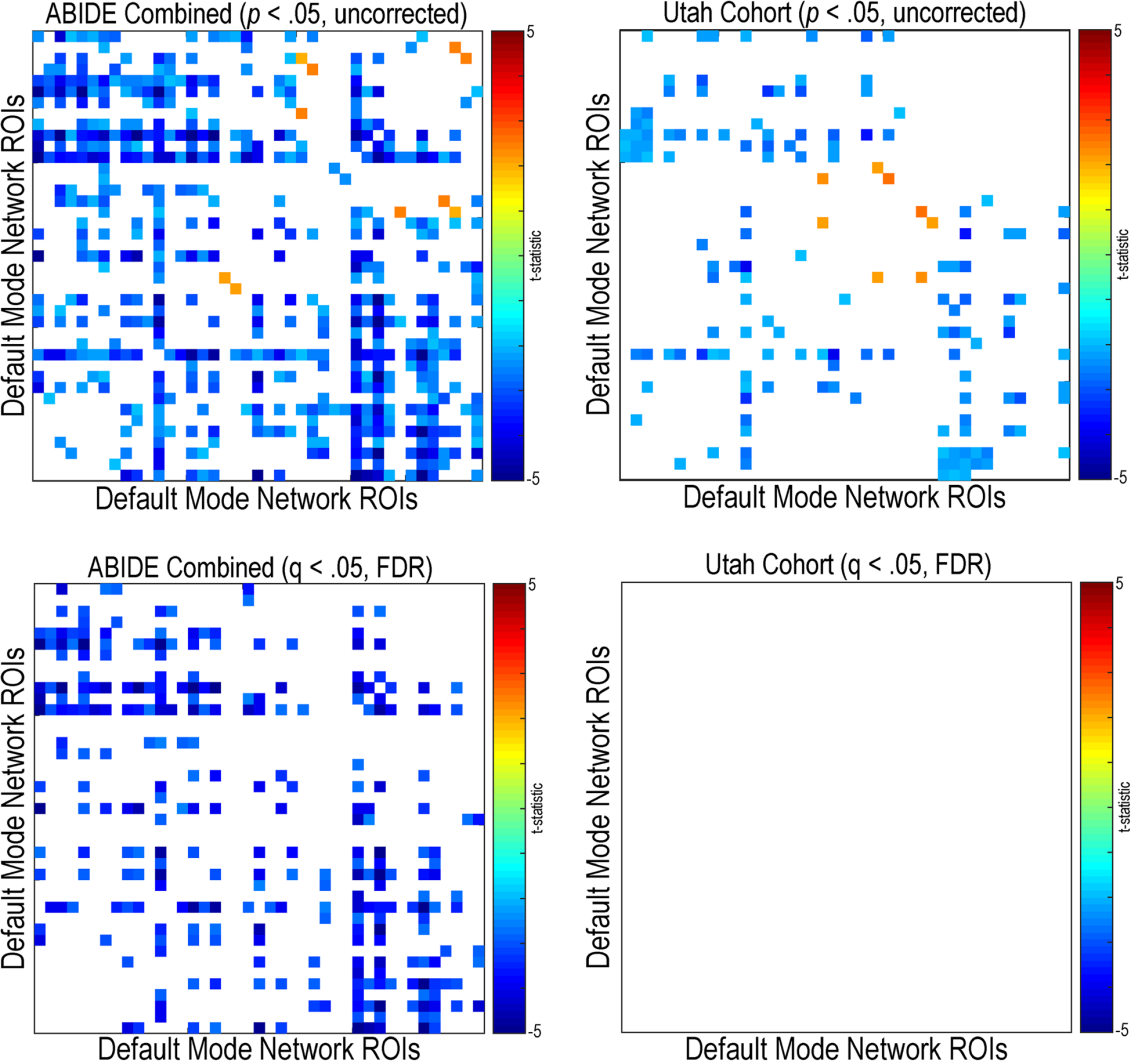
Within default mode network resting-state functional connectivity in autism. Functional connectivity was calculated for 41 region pairs making up the default mode network for the combined ABIDE dataset, and a high-temporal resolution replication sample (Utah cohort). Between-group differences were calculated using a general linear model controlling for age, sex, mean head motion, and site in the combined ABIDE dataset, and age and mean head motion in the Utah cohort. Uncorrected findings are represented on the top panel (*p* < .05, uncorrected). Multiple comparison findings are represented on the bottom (*q* (FDR) < .05). Cooler colors represent autism < controls

**Fig. 11:**
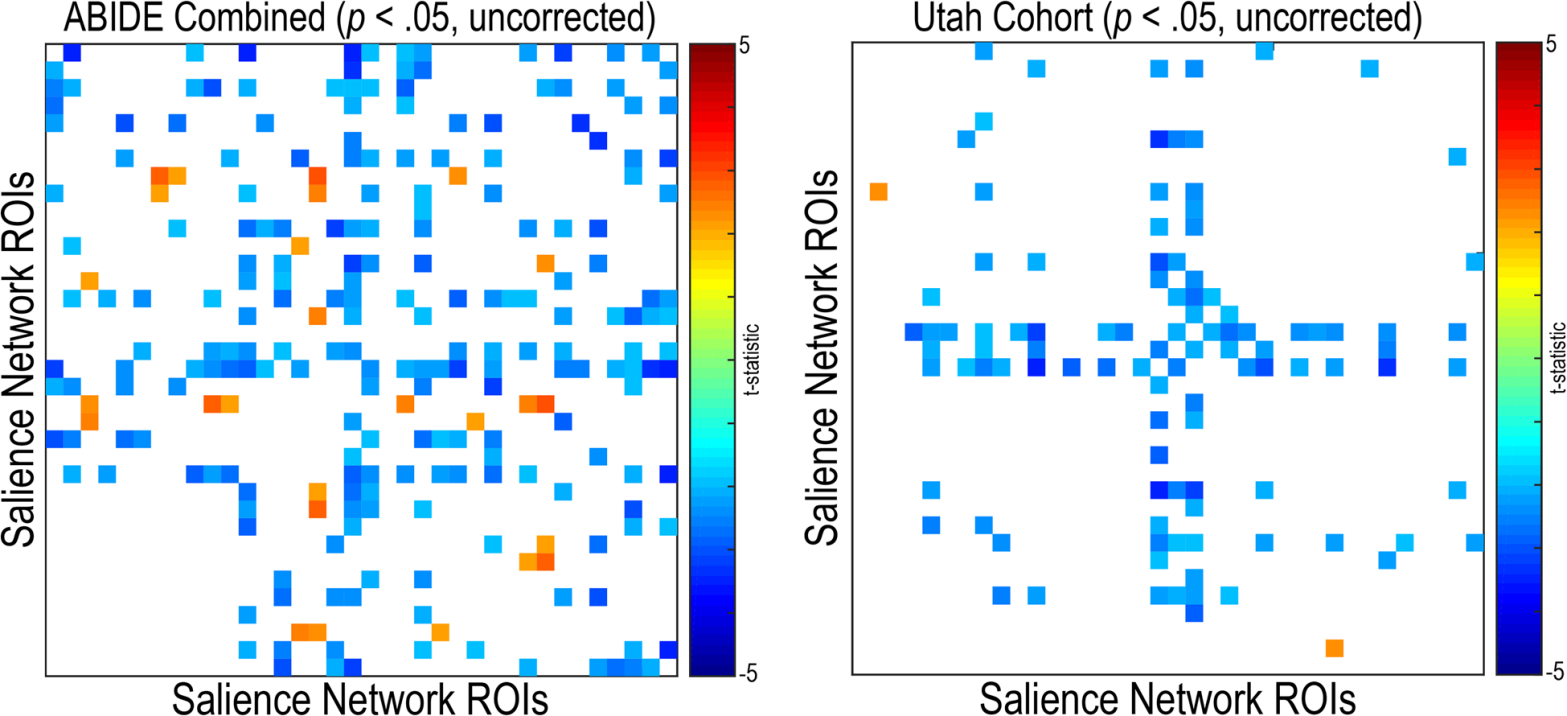
Within salience network resting-state functional connectivity in autism. Functional connectivity was calculated for 36 region pairs making up the salience network for the combined ABIDE dataset, and a high-temporal resolution replication sample (Utah cohort). Between-group differences were calculated using a general linear model controlling for age, sex, mean head motion, and site in the combined ABIDE dataset, and age and mean head motion in the Utah cohort (*p* < .05, uncorrected). Cooler colors represent autism < controls

**Fig. 12:**
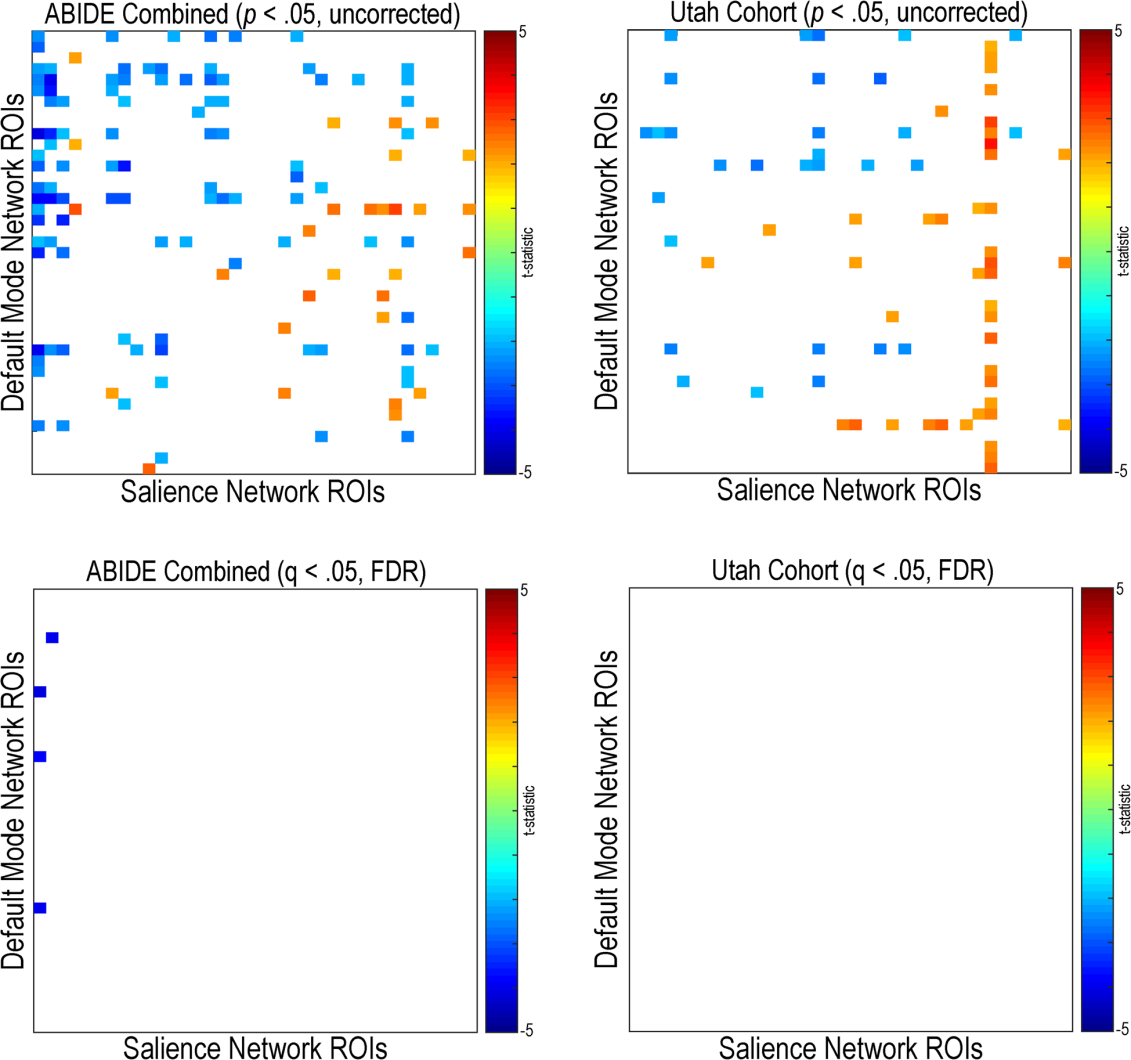
Resting-state functional connectivity between the default mode and salience networks in autism. Functional connectivity was calculated between 36 region pairs making up the salience network and 41 region pairs making up the default mode network for the combined ABIDE dataset, and a high-temporal resolution replication sample (Utah cohort). Between-group differences were calculated using a general linear model controlling for age, sex, mean head motion, and site in the combined ABIDE dataset, and age and mean head motion in the Utah cohort. Uncorrected findings are represented on the top panel (*p* < .05, uncorrected). Multiple comparison findings are represented on the bottom (*q* (FDR) < .05). Cooler colors represent autism < controls

Functional connectivity within the default mode network was primarily decreased in individuals with autism compared to controls for the combined ABIDE sample (*q* (FDR) < .05) (see Fig. 10). Uncorrected findings were replicated in the Utah cohort; however, no findings met multiple comparison correction. Sparse decreased connectivity (*q* (FDR) < .05) within the default mode network was found in individuals with autism compared to controls in Trinity (A1), UM (A1), USM (A1), and ONRC (A2). No significant within salience network findings were found in any of the research sites, the combined ABIDE sample, or the Utah cohort (*q* (FDR) < .05) (see Fig. 11). Sparse decreased connectivity findings, in individuals with autism compared to controls, were found in the combined ABIDE sample between the default mode and salience networks (q (FDR) < .05) (see Fig. 12). These findings appear to be driven by widespread findings demonstrated in the ONRC (A2) research site (*q* (FDR) < .05).

#### Features

**Fig. 13:**
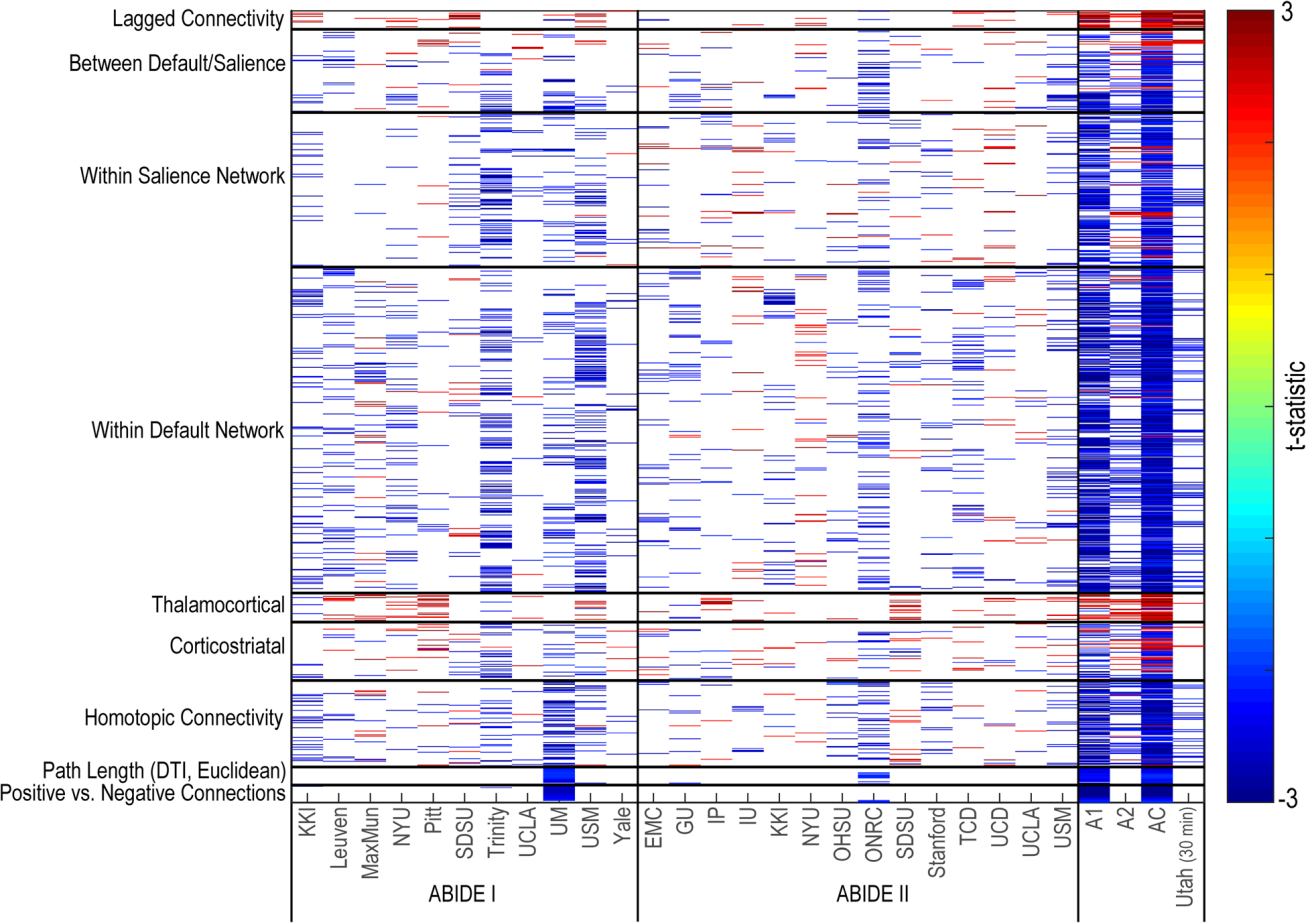
Between-group comparison of resting-state functional connectivity methods. Distribution of between-group resting-state findings for select methods are presented (*p* < .05, uncorrected) for each research site, ABIDE I, ABIDE II, a combined ABIDE dataset, and a high-temporal resolution replication sample (Utah cohort). Cooler colors represent autism < controls

A total of 1229 features met selection criteria and were included in the analysis. Details related to the distribution of the features across methods for each site, ABIDE I, ABIDE II, the combined ABIDE sample, and the Utah cohort replication sample, can be found in Fig. 13 (*q* [FDR] < .05 corrected results can be found in Additional file 1: Figure S8). No single method demonstrated stability across sites including in a high-temporal resolution replication sample (Utah cohort). Nevertheless, patterns were observed in the directionality of site-specific results, with functional connectivity either increased or decreased in individuals with autism for the majority of sites for a given analysis method, with relatively few exceptions. However, the strong disparity in result reproducibility between ABIDE I and ABIDE II suggests poor generalizability across research sites and similarities across features in which participants are identified by the feature. In nearly all features showing *p* < .05 for the Utah high-temporal resolution replication sample, the direction of effect was the same as for the combined ABIDE sample. In order to identify similarities in findings across sites, results were also sorted by age, mean head motion, or eye status (open/closed) (Additional file 1: Figures S9–S11). For all three cases, reordering the sites did not provide a strong explanation for sources of limited reproducibility. Replacing mean head motion with percent motion-free volumes did not significantly impact the results.

**Fig. 14:**
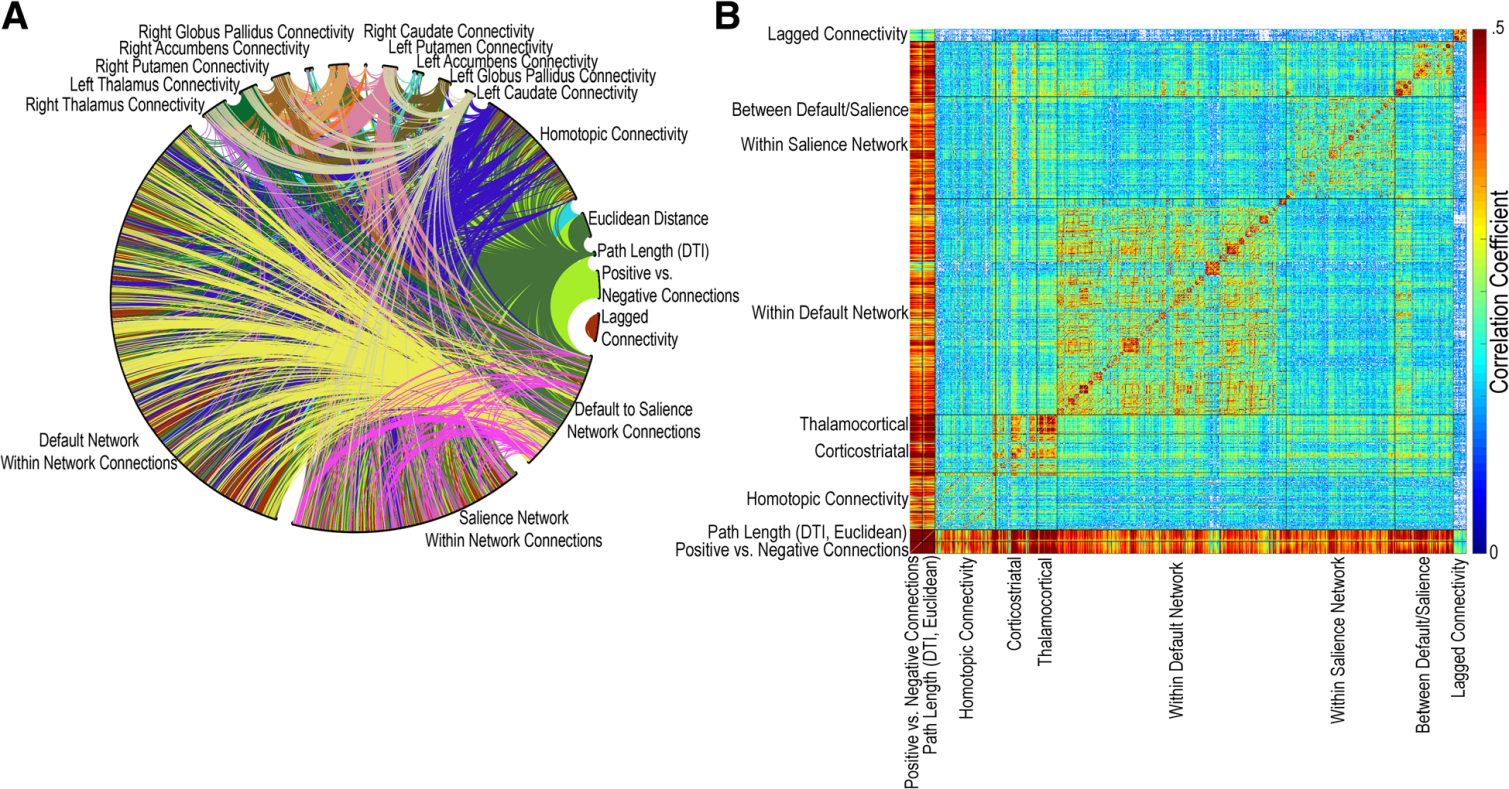
Resting-state functional connectivity methods in autism. **a** Circular graph representing the correlation across individuals with autism of resting-state methods in the combined ABIDE autism sample. Lines are drawn between two features if the absolute value of the Pearson correlation coefficient across subjects for the two features is greater than 0.4. **b** Correlation of features across participants for select resting-state functional connectivity methods in the combined ABIDE autism sample (*q* (FDR) < .05)

Using this set of 1229 features, we investigated whether similar subjects showed relatively higher or lower connectivity for each pair of features. It remains unclear in the literature whether different types of analysis of functional connectivity data similarly identify the same cohorts of subjects that drive results, or which types of analysis exhibit the highest similarity across subjects. Moreover, do abnormalities in specific types of analysis such as homotopic connectivity, corticostriatal connectivity, or long-range connectivity tend to identify different groups of subjects, which would be expected if these different types of analysis targeted distinct aspects of autism pathophysiology. To visualize these data, we calculated the Pearson correlation coefficient between each pair of features across all 1402 subjects in the ABIDE combined sample. Figure 14 demonstrates how features exhibit a dense web of interconnections representing a similar distribution across participants identified by the features for all but one feature type (lag-based connectivity). Correlation values across subjects for each pair of features are shown in Fig. 14 (right).

#### Correlation with autism symptoms

**Fig. 15:**
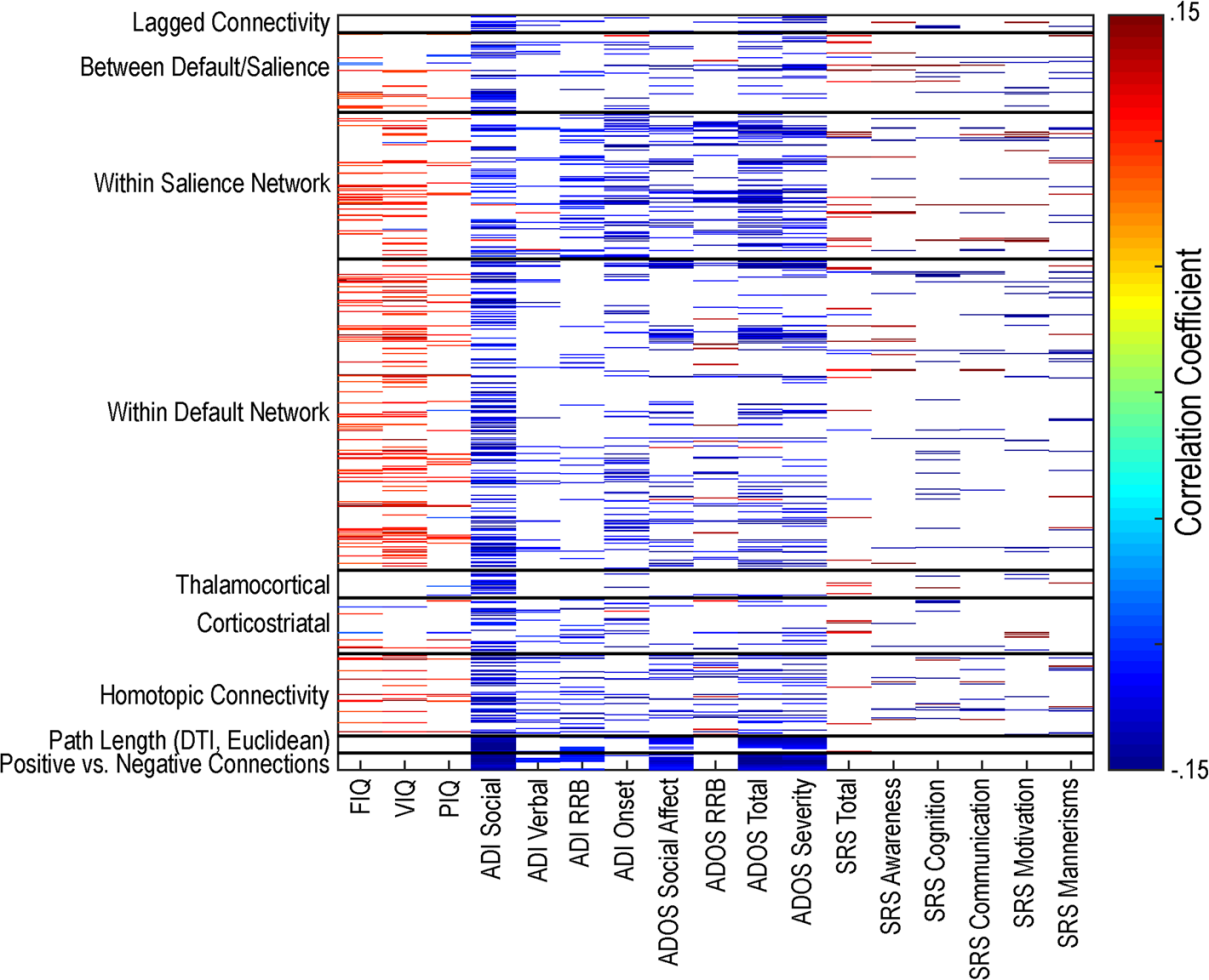
Correlations between behavioral measures and resting-state functional connectivity methods features. Correlations between behavioral scores and 1229 features (*p* < .05) for the combined ABIDE autism sample. VIQ = verbal IQ; PIQ = performance IQ; FIQ = full-scale IQ; ADI = Autism Diagnostic Interview; ADOS = Autism Diagnostic Observation Schedule; SRS = Social Responsiveness Scale

Correlations were calculated between all 1229 features and available behavioral measures in the combined ABIDE autism sample (see Additional file 1: Table S4 for information related to behavioral measures between groups). Correlations between measures of IQ and functional connectivity method results were primarily positive while measures related to autism symptoms were primarily negative (see Fig. 15). From these data, it appears that distinct types of functional connectivity analysis such as corticostriatal connectivity, homotopic connectivity, or within default network connectivity do not clearly segregate with specific types of autism symptoms. Rather, it appears that features most related to different aspects of autism symptoms are distributed across analysis methods without a clear underlying pattern.

**Fig. 16:**
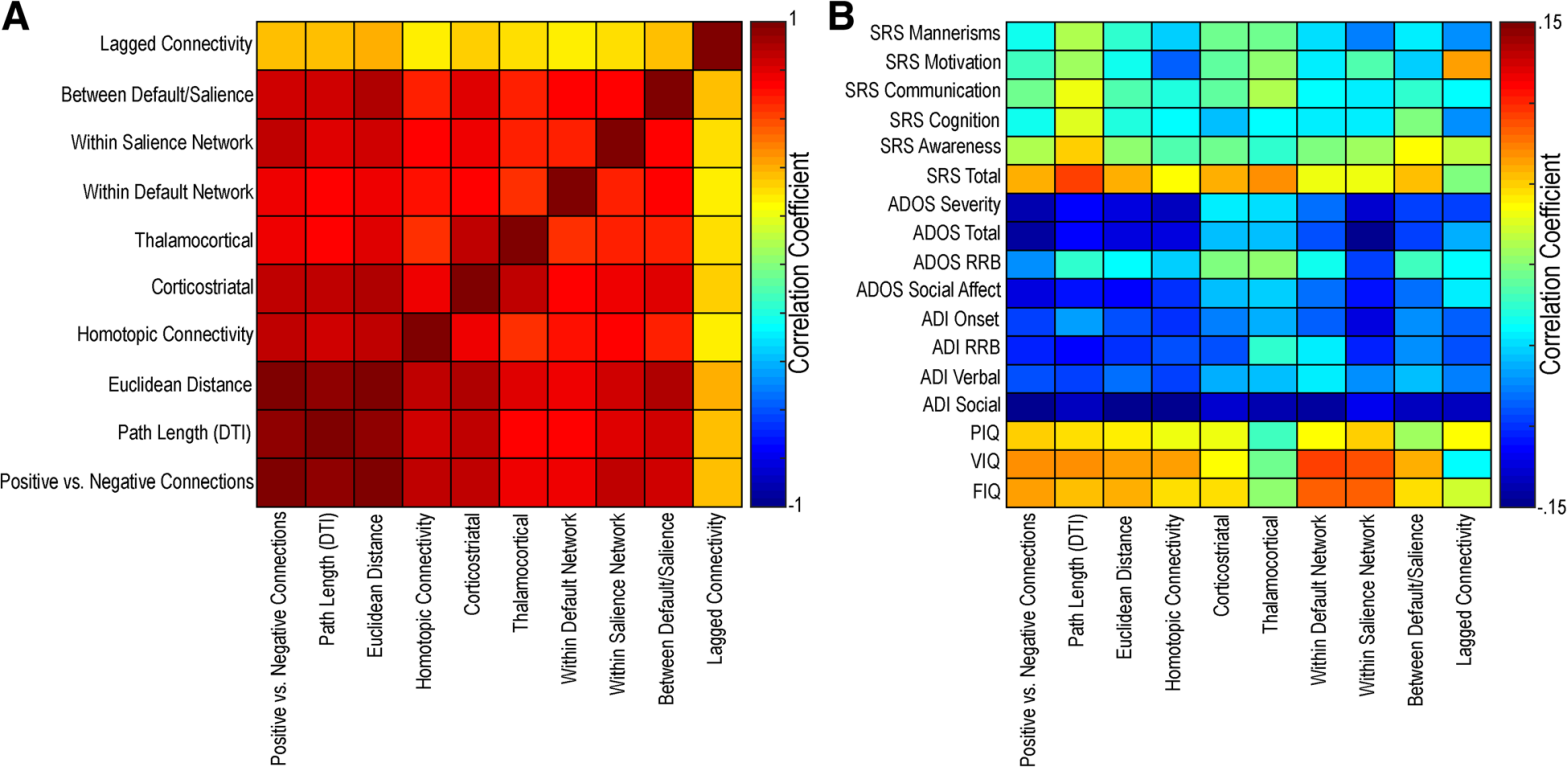
Correlation between averaged resting-state functional connectivity methods and behaviors. **a** Correlation between feature types across participants (unthresholded). **b** Correlation between feature types and behavioral measures (unthresholded). VIQ = verbal IQ; PIQ = performance IQ; FIQ = full-scale IQ; ADI = Autism Diagnostic Interview; RRB = Restricted and Repetitive Behaviors;  ADOS = Autism Diagnostic Observation Schedule; SRS = Social Responsiveness Scale

It is possible that limited reproducibility across sites arises from variability inherent in the features (noise) rather than from true heterogeneity across individuals, particularly given that the datasets from ABIDE are largely obtained from acquisition methods with limited resolution and duration (<=10 min per participant). In order to investigate differences in the relationship between methods and behavioral measures, features were averaged within each method type and again correlated with behaviors. Methods based on static functional connectivity were again strongly correlated with each other; however, lagged-based functional connectivity was not strongly correlated with the other measures suggesting it may measure different aspects of brain function in individuals with autism. However, the correlation between functional connectivity methods and behaviors demonstrate that they are all related to autism traits (see Fig. 16).

As an additional investigation into what factors may be related to limited reproducibility, we performed a more systematic investigation of demographic and technical factors that might underlie limited replication of functional connectivity results. We evaluated the combined ABIDE data by removing one site at a time and comparing the site-specific results for that site across features (vector of autism vs. control *t* statistics for 1229 features) to the results from the remaining ABIDE sample, yielding a measurement of “generalizability” for data from each site. We compared this measure using partial correlation across sites to seven factors: age, sex ratio, mean head motion, percent of motion free data, eyes open vs. eyes closed status, temporal resolution (repetition time), and number of subjects per site. Of these, only the number of subjects per site showed significant correlation to generalizability across sites (*r* = .65, *p* = .0024), although the other 6 factors showed variable partial correlation across sites that may contribute to limited reproducibility (see Additional file 1: Figure S12). Nevertheless, almost all of the data in the ABIDE sample was acquired with temporal resolutions of at least 2 s, and it is possible that faster acquisitions that can freeze effects of breathing and mitigate aliasing of heart rate artifacts may offer nontrivial improvement in results.

## Discussion

This study examined whether different aspects of functional MRI connectivity described in the literature represent distinct symptoms of autism or cohorts of individuals with autism and the extent to which these functional connectivity methods exhibit reproducibility across individuals and datasets. For all functional connectivity methods tested, results showed poor generalizability across sites and participants rather than clear reproducibility, with no method demonstrating highly reproducible results when compared to the entire multi-site ABIDE sample (see Fig. 13 for summary figure with *p* < .05 (uncorrected) results; (*q* [FDR] < .05 corrected results can be found in Additional file 1: Figure S8). When functional connectivity features were compared to behavioral symptoms, distinct types of analysis, such as corticostriatal, homotopic, or default mode connectivity, did not correlate with different types of autism symptoms. Rather, individual connections or features that tracked with different symptoms of autism were distributed across methodological approaches without any clear pattern.

### Literature comparisons

In the current study, few sites demonstrated significant between-group differences in positive vs. negative functional connectivity assessed using bins of connections with similar connectivity in the independent Human Connectome Project sample. Short-range and long-range connectivity results were also inconsistent across sites, consistent with a recent analysis demonstrating only region-specific local overconnectivity using a regional homogeneity approach, with different subgroups of subjects demonstrating variably higher or lower long-distance connectivity [62]. Variability in local connectivity has also been demonstrated with cohorts differing in fMRI acquisitions with eyes open vs. eyes closed [63]. Theoretical proposals of long-range under-connectivity and short-range over-connectivity [1, 18–20] have been variably defined in terms of distance ranging from cortical columns to many centimeters, and studies examining distant connections have produced variable over- and under-connectivity. The analysis in the current sample may be limited by the use of an independent dataset (Human Connectome Project) not matched for sex and age to define distances.

Encouragingly, the current study found decreased homotopic connectivity which has been reported and replicated in the literature [28, 29]. Though there were some sites that demonstrated increased homotopic connectivity, findings reaching multiple comparison correction were nearly all decreased in individuals with autism compared to controls.

Consistent with the literature, this study also found both hypo- and hyper-connectivity in corticostriatal connections [3–7]; however, the direction of these findings was not consistent between research sites, and it appears to be predominantly decreased in individuals with autism compared to controls when larger sample sizes are assessed. Similar incongruities were found with thalamocortical connectivity which also varies in the literature with respect to directionality [4, 9].

In the current study, idiosyncrasy was estimated by calculating the variance between an individual’s time series data for each ROI and an averaged value based on an independent dataset. Two sites showed decreased idiosyncrasy in individuals with autism compared to controls. This finding is in contrast to outcomes in the literature that report increased idiosyncrasy in individuals with autism compared to controls [26, 27]. This inconsistency is likely attributable to methodological differences as the majority of studies in the literature utilize machine learning techniques to establish idiosyncratic values.

Similarly, the current study found no between-group differences in modularity for any research site or combined dataset; however, increased global efficiency was found in individuals with autism compared to controls for two research sites. Both increased and decreased global efficiency in individuals with autism compared to controls have been demonstrated in the literature [21, 22]. With regard to within and between default mode and salience networks, the findings in the current study closely mirror those from the literature [3, 10–15]; however, it is important to note that few research sites demonstrated multiple comparison corrected findings (see Additional file 1). Indeed, widespread decreased connectivity meeting FDR correction was only evident in the larger combined ABIDE dataset with respect to inter-default mode network connectivity.

Overall, we found poor generalizability across sites when testing which functional connectivity features predict autism, with consistent results only for samples of hundreds of participants. Furthermore, different types of functional connectivity features (homotopic, thalamocortical, corticostriatal, specific networks) seem to not consistently predict different features of autism. Rather, specific features that predict autism symptoms seem to be distributed across feature types. Interestingly, there is a web of interrelationships between which features are high in which participants, with only lagged connectivity not showing correlation across individuals with autism with other feature types. As more features are added together, consistent results are obtained regardless of which feature types are added. It may be that these findings reflect global connectivity, which predicts ADOS and ADI scores but not SRS scores. Indeed, measures of global connectivity have been found to decrease in individuals with autism compared to controls [34, 64]. Even when using modern acquisition strategies (multi-band data, 30-min acquisition times per participant), heterogeneity and modest prediction rates for autism are seen, although findings were very consistent with those obtained from the entire ABIDE sample, suggesting that long-duration, high-temporal resolution acquisitions may improve replicability of results. Holiga and colleagues used data from 4 separate datasets including ABIDE I and ABIDE II and examined reproducibility of degree centrality as a metric distinguishing autism from control individuals [8]. While effect sizes were large in the EU-AIMS and InFoR datasets (Cohen’s *d* > .8 for some measures), effect sizes were much smaller in ABIDE datasets (*d* = .2), possibly indicating that technical parameters of acquisition may contribute to reproducibility of the results, given that EU-AIMS data were acquired with more volumes and using a multi-echo technique. Similarly, a recent report imaging participants with autism and low cognitive and verbal performance identified similar connectivity differences to the entire ABIDE sample in this report within a single site’s data [65]. While none of the individual features tested show promise in this analysis as sensitive and specific biomarkers, consistent with recent reviews [66, 67], the individual features demonstrated a rich web of interrelationships across subjects as well as differences across subjects that may inform efforts to identify clinical subtypes [68] or use multi-parametric deep-learning approaches to arrive at more sensitive and specific imaging markers [69].

### Limitations

A number of limitations should be considered. Though we consider all participant data being processed using the same parameters a strength of this study, certain aspects of that model could act as a confound. For example, differences in acquisition parameters, volume numbers, and spatial scale may benefit from preprocessing pipelines more suited to the nuances of each study site with the ABIDE dataset. Second, while efforts were made to minimize variance due to differences in the research site, statistical controls are likely not able to account for more nuanced between-site variance. Third, while we attempted to replicate methods identified in the literature, all method tests were based on a common parcellation scheme that was created using imaging data from adult participants. Many of the participants included in the ABIDE dataset are children or adolescents. Thus, it may be that lack of reproducibility across methods reported in this study are tied to differences in cortical parcellations, nuanced atlas registration, or changes across development; though, many of the methods tested do not require extremely precise cerebral region assignment (long-range vs. short-range, positive vs. negative, etc.), and age was included as a covariate in all analyses. Finally, it cannot be overstated that strategic choices in image postprocessing have a clear effect on functional connectivity results [70], and different choices in postprocessing may have resulted in improved or poorer reproducibility.

## Conclusions

Functional connectivity in autism is characterized more by variability than consistent reproducibility across participants and sites, which may be attributed to multi-factorial demographic and technical differences including recruitment strategy across autism and neurotypical cohorts, age of participants, male to female ratios, duration of acquisition, and technical acquisition parameters. Different functional connectivity methods did not identify distinct behavioral correlations for participant cohorts; however, most functional connectivity methods covary with ADOS, ADI, and SRS scores, indicating a relationship to autism symptomatology. We did not observe that different approaches to functional connectivity (e.g., homotopic connectivity, long-range vs. short-range connectivity, corticostriatal connectivity), nor connections associated with distinct spatial regions or networks, cleanly identify with different behavioral features of autism. Extensive variability and limited reproducibility were not overcome by averaging results of many features of a similar type or by extensive postprocessing to mitigate physiological and technical artifacts. Many functional connectivity features do show differences in symptom profiles; methods from deep-learning and big data analysis, combined with large feature sets from functional connectivity data, may be promising approaches to prognosis, outcomes monitoring, and treatment effects.

## Additional file

Additional file 1: Supplementary Information results. (PDF 8812 kb)

## Abbreviations

ABIDE: Autism Brain Imaging Data Exchange
ADI: Autism Diagnostic Interview-Revised
ADOS: Autism Diagnostic Observation Schedule
AFNI: Analysis of Functional NeuroImages
BOLD: Blood-oxygen-level-dependent
DTI: Diffusion tensor imaging
EEG: Electroencephalography
FDR: False discovery rate
fMRI: functional magnetic resonance imaging
IQ: Intelligence quotient
MEG: Magnetoencephalography
ME-ICA: Multi-echo independent component analysis
MRI: Magnetic resonance imaging
ROI: Region of interest
SRS: Social Responsiveness Scale
