# Supplementary material for: Generalizability and reproducibility of functional connectivity in autism
Source: Mol Autism. 2019 Jun 24;10:27. doi: 10.1186/s13229-019-0273-5 (PMC6591952; doi:10.1186/s13229-019-0273-5)
Supplement: Supplementary file 1 — Supplementary Information results. (PDF 8812 kb) [file 13229_2019_273_MOESM1_ESM.pdf]

Supplementary Information

Additional File 1. Supplementary Information Results

Supplementary Table 1. List of research sites included in the ABIDE dataset

**ABIDE I Sites**	
KKI	Kennedy Krieger Institute
Leuven	University of Leuven
MaxMun	Ludwig Maximilian University of Munich
NYU	New York University Langone Medical Center
Pitt	University of Pittsburgh School of Medicine
SDSU	San Diego State University
Trinity	Trinity Centre for Health Sciences
UCLA	University of California Los Angeles
UM	University of Michigan
USM	University of Utah School of Medicine
Yale	Yale Child Study Center
**ABIDE II Sites**	
EMC	Eramus University Medical Center
GU	Georgetown University
IP	Institut Pasteur and Robert Debré Hospital
IU	Indiana University
KKI	Kennedy Krieger Institute
NYU	New York University Langone Medical Center
OHSU	Oregon Health and Science University
ONRC	Olin Neuropsychiatry Research Center, Institute of Living at Hartford Hospital
SDSU	San Diego State University
Stanford	Stanford University
TCD	Trinity Centre for Health Sciences
UCD	University of California Davis
UCLA	University of California Los Angeles
USM	University of Utah School of Medicine

Supplementary Table 2. Demographic information for each research site in the ABIDE I dataset

ABIDE I by Site												
	***N* =**			**Male % (total sample)**			**Age (SD)**			**Motion (SD)**		
	**ASD**	**TD[a]**	**Total**	**ASD**	**TD**	**Total**	**ASD**	**TD**	***P***	**ASD**	**TD**	***P***
KKI	12	30	42	21.4	50.0	71.4	9.7±1.3	10.2±1.3	.314	.14±.10	.11±.04	.285
Leuven	22	32	54	37.0	51.9	88.9	18.9±5.2	18.6±5.0	.831	.11±.04	.11±.03	.856
MaxMun	14	24	38	28.9	55.3	84.2	26.9±13.9	27.0±10.5	.994	.09±.08	.10±.05	.641
NYU	72	104	176	35.2	44.3	79.5	14.7±7.1	15.9±6.2	.258	.09±.04	.08±.04	.019
Pitt	17	16	33	39.4	42.4	81.8	18.5±7.7	17.4±4.6	.601	.14±.04	.14±.03	.684
SDSU	10	20	30	33.3	46.7	80.0	14.8±1.7	14.1±2.0	.398	.07±.04	.08±.04	.647
Trinity	20	21	41	48.8	51.2	100.0	17.1±2.8	17.4±3.8	.759	.12±.03	.10±.02	.012
UCLA	23	28	51	41.2	45.1	86.3	14.3±2.5	13.5±1.9	.231	.09±.05	.09±.07	.971
UM	45	72	117	31.6	47.0	78.6	13.5±2.3	14.8±3.6	.018	.11±.06	.09±.06	.089
USM	46	38	84	54.8	45.2	100.0	22.6±7.5	21.7±7.6	.595	.11±.05	.11±.04	.561
Yale	14	13	27	40.7	37.0	77.8	12.6±3.2	12.9±3.1	.783	.14±.05	.10±.04	.010

[a]Typically Developing controls

Supplementary Table 3. Demographic information for each research site in the ABIDE II dataset

ABIDE II by Site												
	***N* =**			**Male % (total sample)**			**Age (SD)**			**Motion (SD)**		
	**ASD**	**TD[a]**	**Total**	**ASD**	**TD**	**Total**	**ASD**	**TD**	***P***	**ASD**	**TD**	***P***
EMC	15	21	36	36.1	47.2	83.3	8.6±1.2	8.2±1.0	.212	.08±.03	.13±.08	.013
GU	26	30	56	41.1	32.1	73.2	11.1±1.6	10.2±1.7	.045	.14±.07	.11±.04	.057
IP	12	25	37	24.3	21.6	45.9	16.1±5.4	25.3±10.3	.001	.09±.05	.06±.02	.084
IU	14	18	32	37.5	43.8	81.3	25.8±11.0	23.9±5.1	.570	.09±.03	.09±.03	.486
KKI	32	122	154	14.3	52.6	66.9	10.3±1.4	10.4±1.2	.799	.14±.05	.11±.05	.042
NYU	36	25	61	52.5	37.7	90.2	10.6±6.3	9.6±3.6	.487	.13±.05	.10±.05	.017
OHSU	33	54	87	29.9	29.9	59.8	11.8±2.4	10.4±1.6	.007	.12±.05	.10±.05	.161
ONRC	15	32	47	29.8	40.4	70.2	21.3±4.0	23.9±3.5	.028	.10±.03	.10±.04	.521
SDSU	31	24	55	43.6	40.0	83.6	13.1±3.3	13.3±3.1	.764	.09±.05	.08±.05	.540
Stanford	18	19	37	45.9	45.9	91.9	11.2±1.2	11.0±1.3	.600	.09±.04	.08±.04	.888
TCD	14	18	32	43.8	56.3	100.0	15.3±3.5	16.3±2.8	.367	.14±.06	.11±.05	.193
UCD	16	11	27	48.1	25.9	74.1	14.9±2.0	15.2±1.5	.692	.11±.06	.09±.03	.246
UCLA	11	12	23	47.8	34.8	82.6	12.2±1.7	10.0±2.3	.013	.10±.03	.10±.06	.881
USM	11	14	25	36.0	44.0	80.0	19.4±8.0	24.0±7.3	.150	.16±.10	.10±.04	.092

[a]Typically Developing controls

Supplementary Table 4. Behavioral information for the combined ABIDE dataset

	Autism		TD[a]		
	N	**Mean (SD)**	***N***	**Mean (SD)**	***P***
VIQ[b]	480	105.70 (17.93)	635	113.85 (13.48)	<.001
PIQ[c]	493	105.18 (17.06)	686	109.43 (13.64)	<.001
FIQ[d]	544	106.05 (16.79)	757	113.03 (12.53)	<.001
ADI[e] Social	379	19.40 (5.57)	0	-	-
ADI Verbal	379	15.40 (4.61)	0	-	-
ADI RRB	380	5.86 (2.52)	0	-	-
ADI Onset	327	3.16 (1.31)	0	-	-
ADOS[f] Social Affect	319	9.43 (3.80)	16	1.13 (1.45)	<.001
ADOS RRB	319	3.10 (1.80)	16	0.31 (0.79)	<.001
ADOS Total	324	12.53 (4.53)	16	1.44 (1.50)	<.001
ADOS Comparison Score	322	7.05 (2.02)	16	1.13 (0.50)	<.001
SRS[g] Raw Awareness	222	11.99 (3.99)	312	4.37 (2.81)	<.001
SRS Cognition	222	16.53 (5.98)	312	3.29 (3.24)	<.001
SRS Communication	222	31.20 (10.68)	312	6.28 (5.73)	<.001
SRS Motivation	222	15.22 (6.08)	312	4.33 (3.70)	<.001
SRS Mannerisms	222	17.80 (6.76)	312	2.48 (2.99)	<.001
SRS Raw Total	352	92.82 (29.72)	432	20.24 (14.96)	<.001

[a]Typically Developing controls; [b]Verbal IQ; [c]Performance IQ; [d]Full-scale IQ; [d]Autism Diagnostic Interview; [f]Autism Diagnostic Observation Schedule; [g]Social Responsiveness Scale

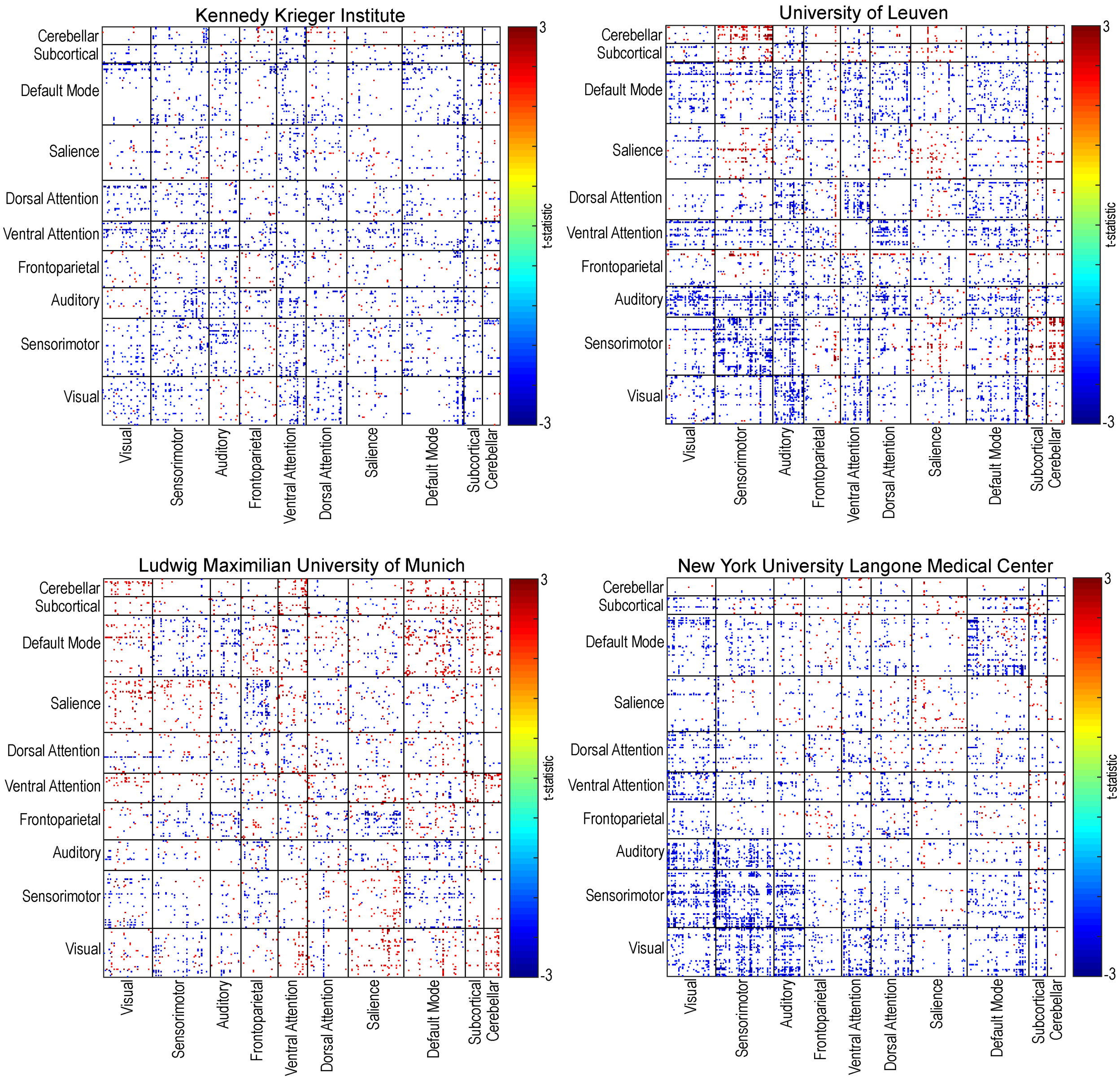

Supplementary Figure 1. ABIDE I resting-state functional connectivity (KKI, Leuven, MaxMun, NYU). Distribution of between-group resting-state findings for a 361 region of interest parcellation in four ABIDE I research sites. Between-group differences were calculated using a general linear model controlling for age, sex, and mean head motion (uncorrected, $p < .05$). Cooler colors represent autism < controls.

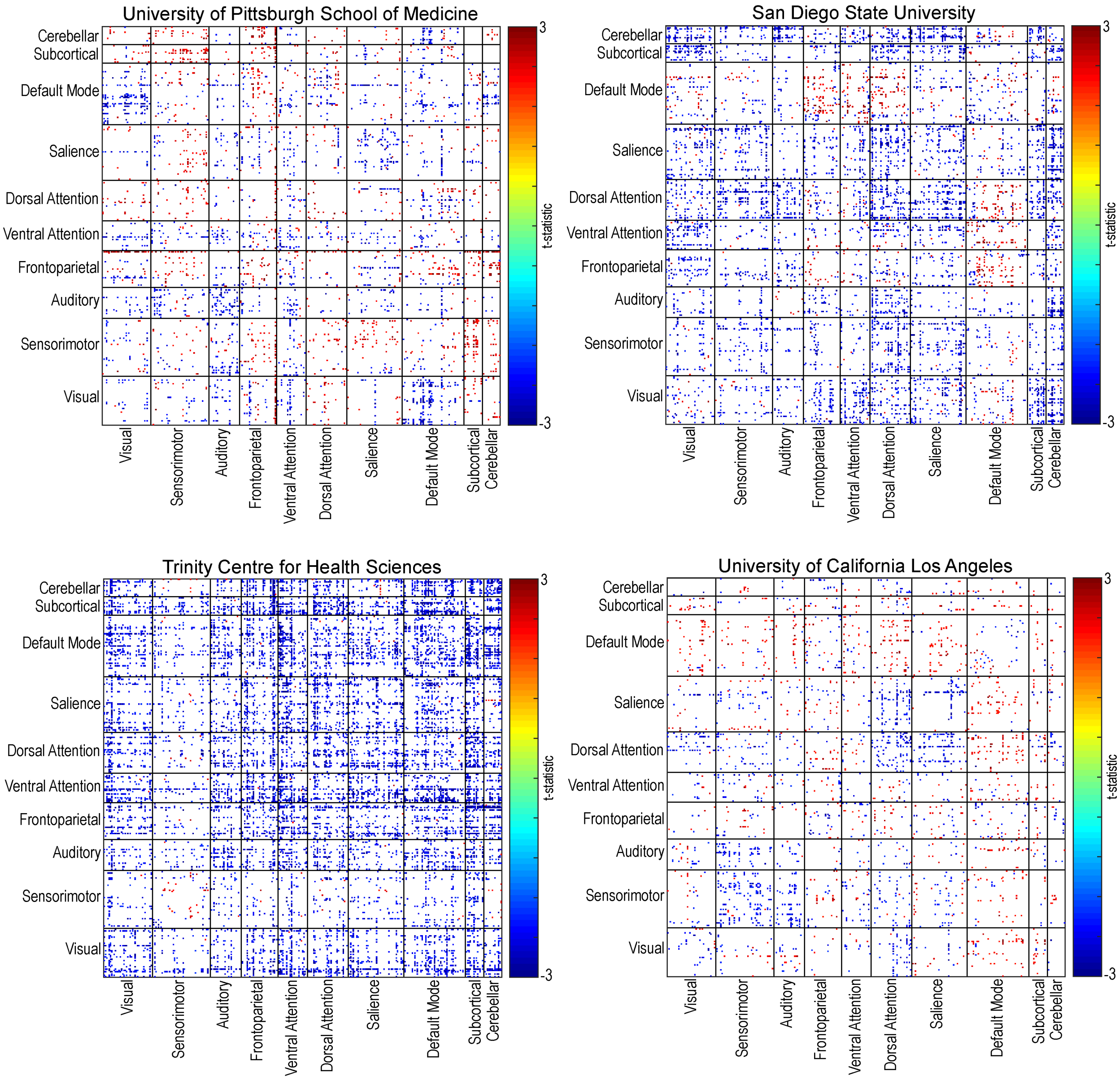

Supplementary Figure 2. ABIDE I resting-state functional connectivity (Pitt, SDSU, Trinity, UCLA). Distribution of between-group resting-state findings for a 361 region of interest parcellation in four ABIDE I research sites. Between-group differences were calculated using a general linear model controlling for age, sex, and mean head motion (uncorrected, $p < .05$). Cooler colors represent autism < controls.

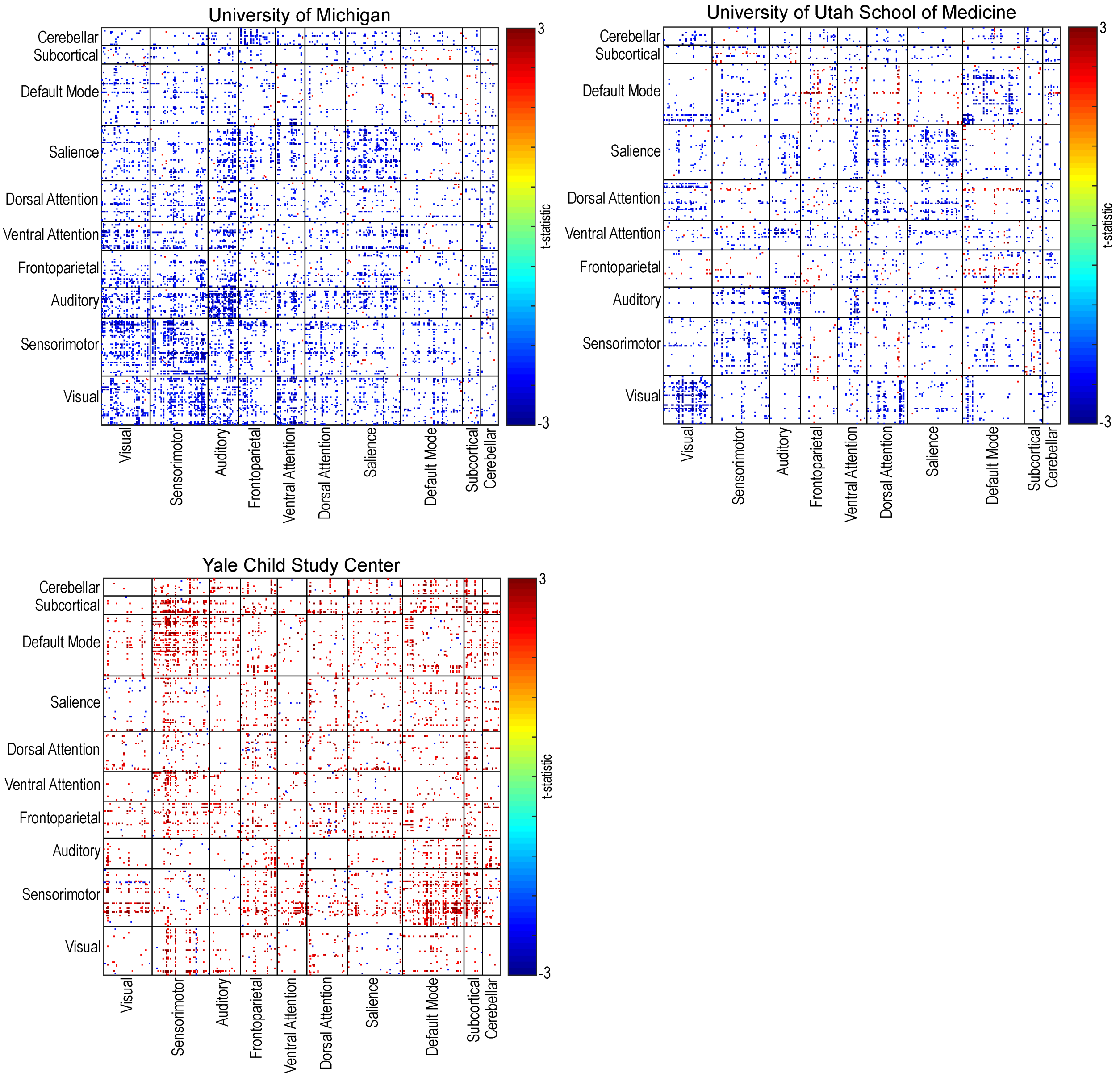

Supplementary Figure 3. ABIDE I resting-state functional connectivity (UM, USM, Yale). Distribution of between-group resting-state findings for a 361 region of interest parcellation in three ABIDE I research sites. Between-group differences were calculated using a general linear model controlling for age, sex, and mean head motion (uncorrected, $p < .05$). Cooler colors represent autism < controls.

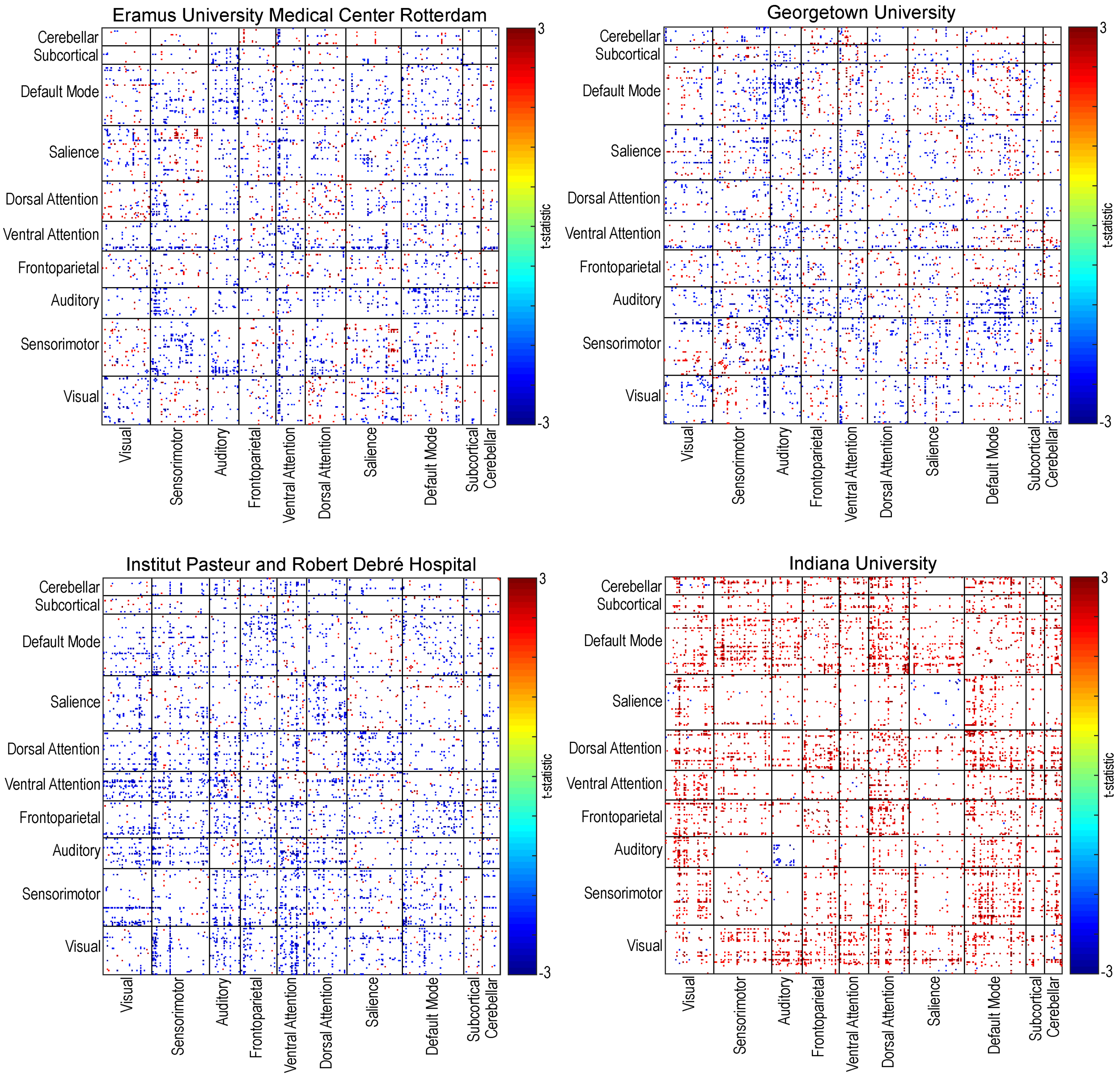

Supplementary Figure 4. ABIDE II resting-state functional connectivity (EMC, GU, IP, IU). Distribution of between-group resting-state findings for a 361 region of interest parcellation in four ABIDE I research sites. Between-group differences were calculated using a general linear model controlling for age, sex, and mean head motion (uncorrected, $p < .05$). Cooler colors represent autism < controls.

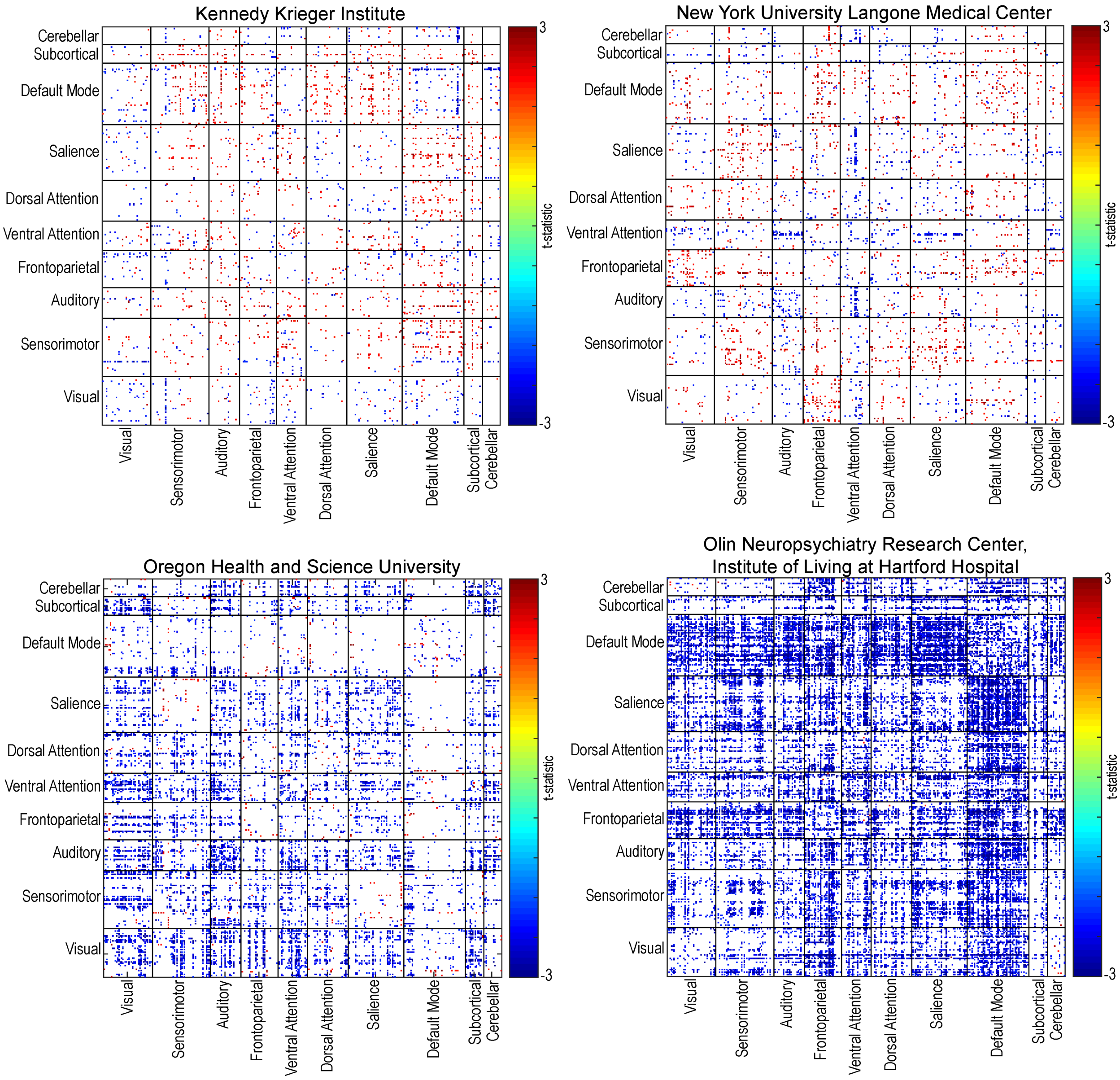

Supplementary Figure 5. ABIDE II resting-state functional connectivity (KKI, NYU, OHSU, ONRC). Distribution of between-group resting-state findings for a 361 region of interest parcellation in four ABIDE I research sites. Between-group differences were calculated using a general linear model controlling for age, sex, and mean head motion (uncorrected, $p < .05$). Cooler colors represent autism < controls.

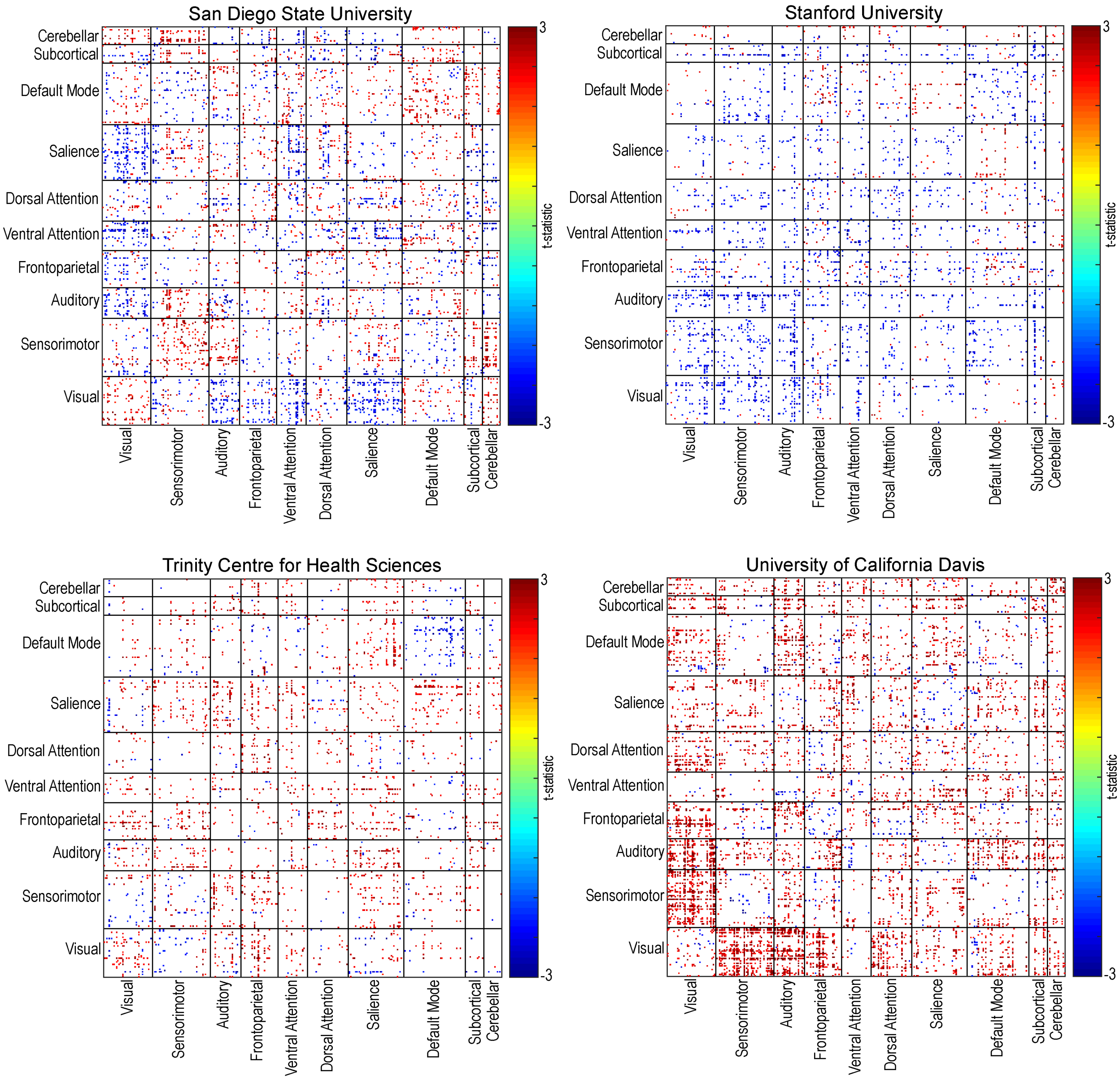

Supplementary Figure 6. ABIDE II resting-state functional connectivity (SDSU, Stanford, TCD, UCD). Distribution of between-group resting-state findings for a 361 region of interest parcellation in four ABIDE I research sites. Between-group differences were calculated using a general linear model controlling for age, sex, and mean head motion (uncorrected, $p < .05$). Cooler colors represent autism < controls.

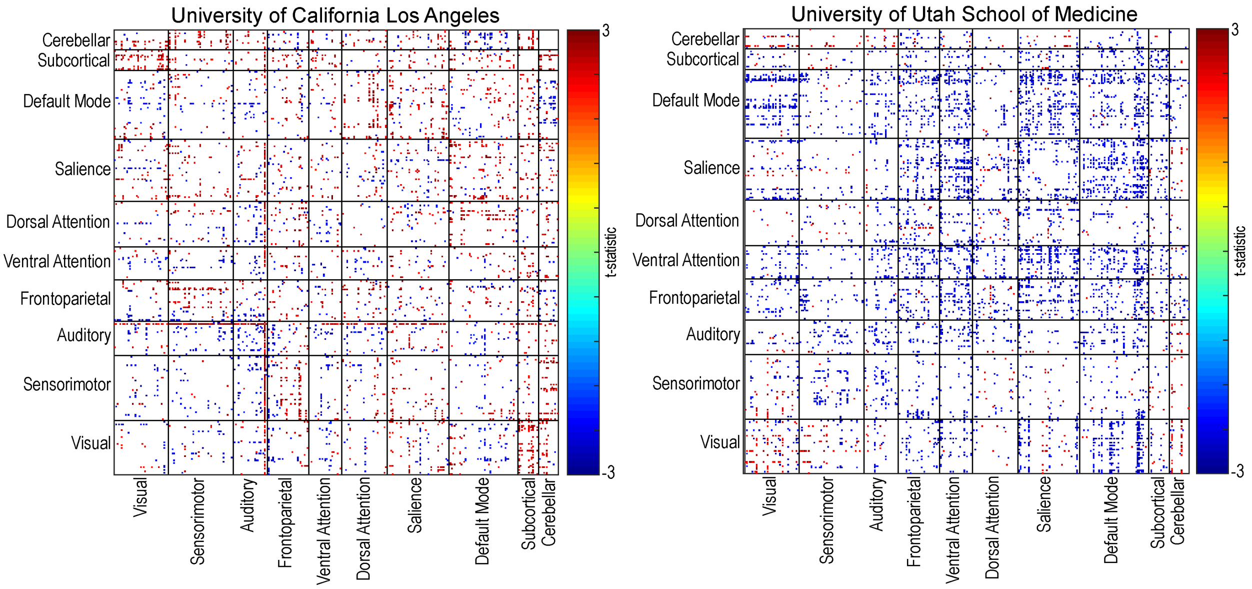

Supplementary Figure 7. ABIDE II resting-state functional connectivity (UCLA, USM). Distribution of between-group resting-state findings for a 361 region of interest parcellation in four ABIDE I research sites. Between-group differences were calculated using a general linear model controlling for age, sex, and mean head motion (uncorrected, $p < .05$). Cooler colors represent autism < controls.

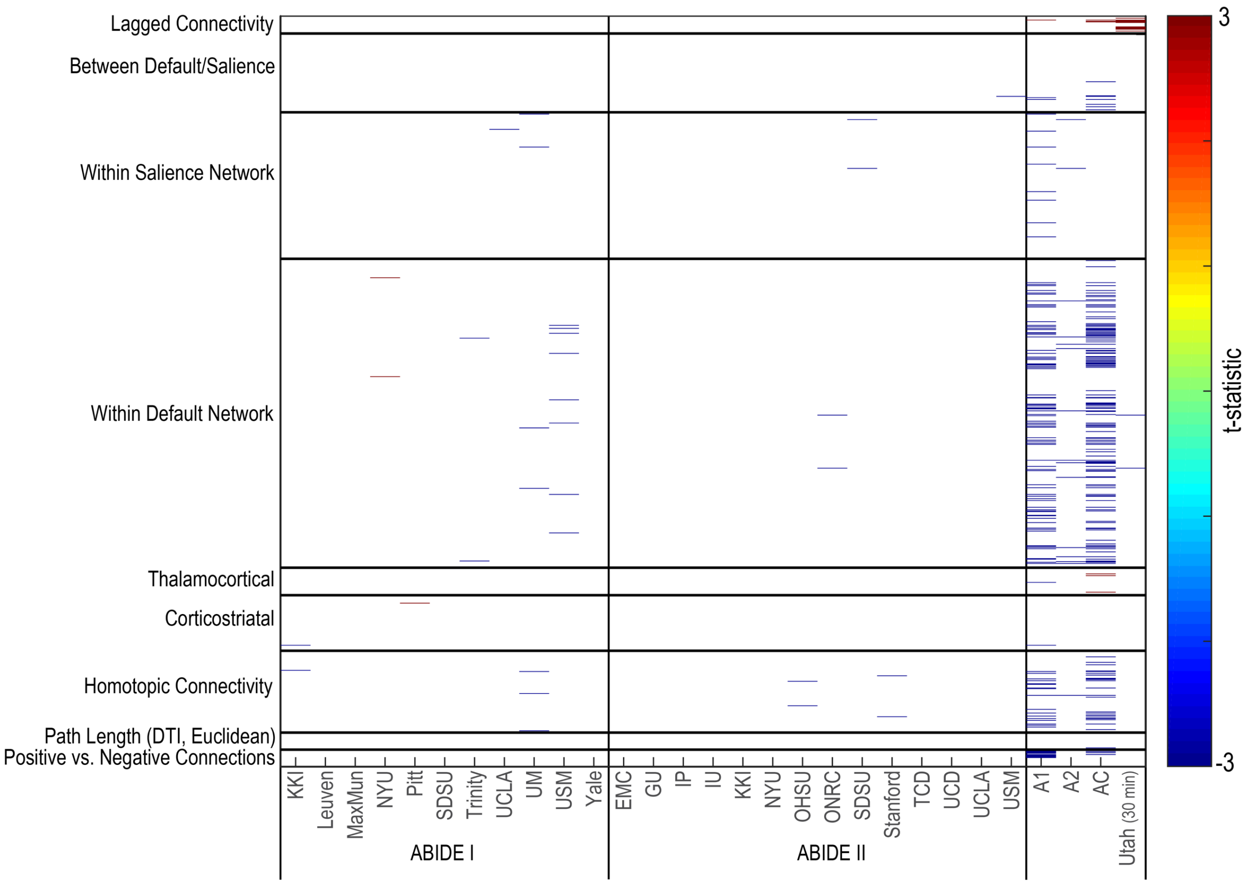

Supplementary Figure 8. Between-group comparison of resting-state functional connectivity methods. Distribution of between-group resting-state findings for select methods are presented (q[FDR] < .05, corrected) for each research site, ABIDE I, ABIDE II, a combined ABIDE dataset, and a high temporal resolution replication sample (Utah cohort). Cooler colors represent autism < controls.

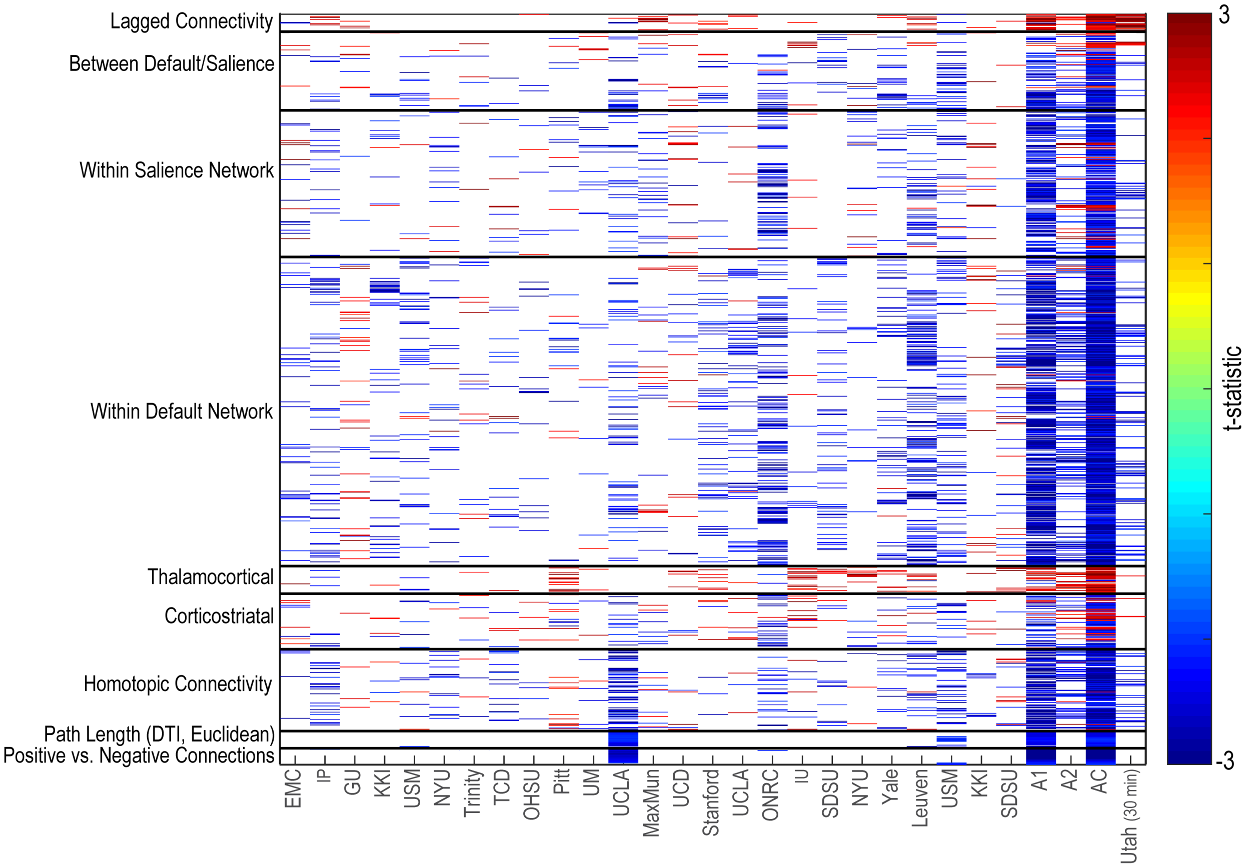

Supplementary Figure 9. Between-group comparison of resting-state functional connectivity methods sorted by age (low to high). Distribution of between-group resting-state findings for select methods are presented (*p* < .05, uncorrected) for each research site, ABIDE I, ABIDE II, a combined ABIDE dataset, and a high temporal resolution replication sample (Utah cohort). Cooler colors represent autism < controls.

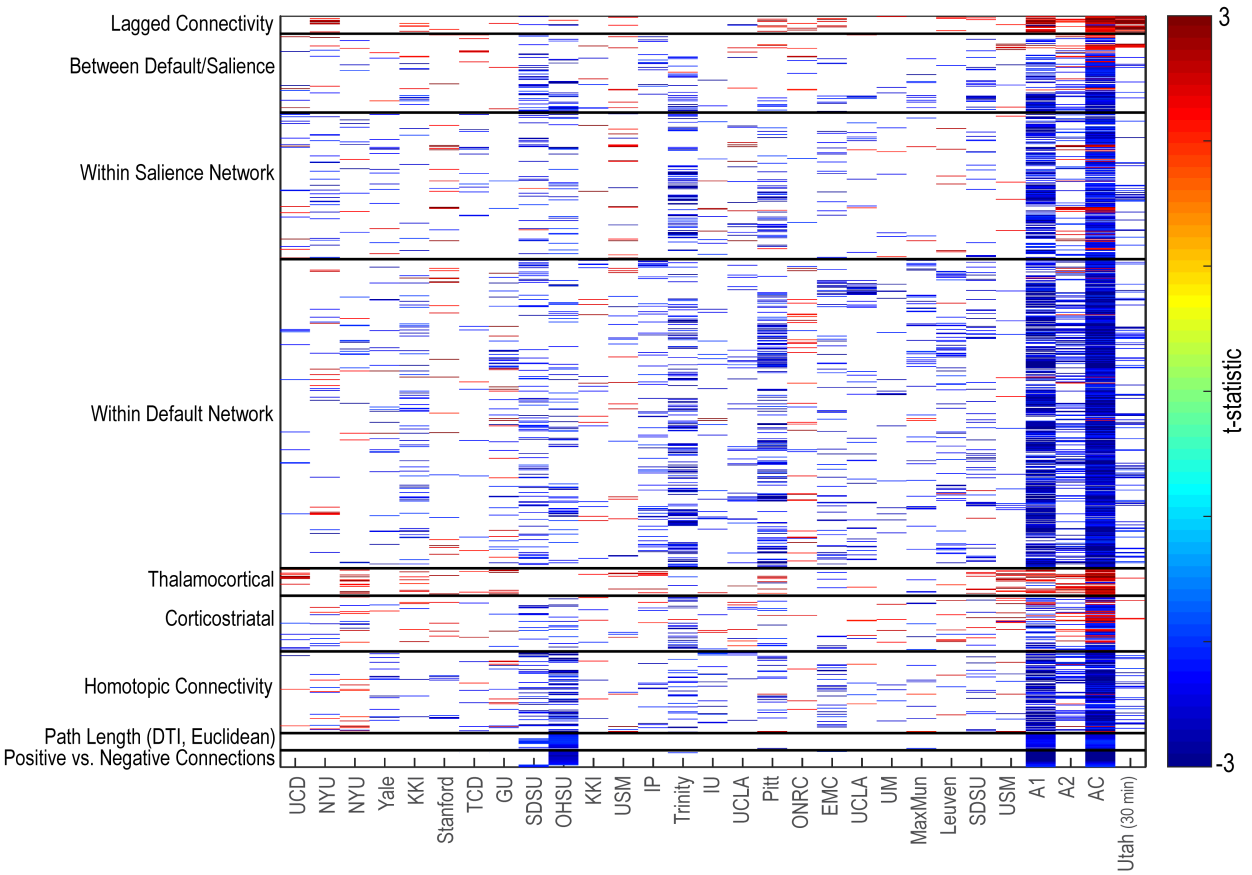

Supplementary Figure 10. Between-group comparison of resting-state functional connectivity methods sorted by motion (low to high). Distribution of between-group resting-state findings for select methods are presented (p < .05, uncorrected) for each research site, ABIDE I, ABIDE II, a combined ABIDE dataset, and a high temporal resolution replication sample (Utah cohort). Cooler colors represent autism < controls.

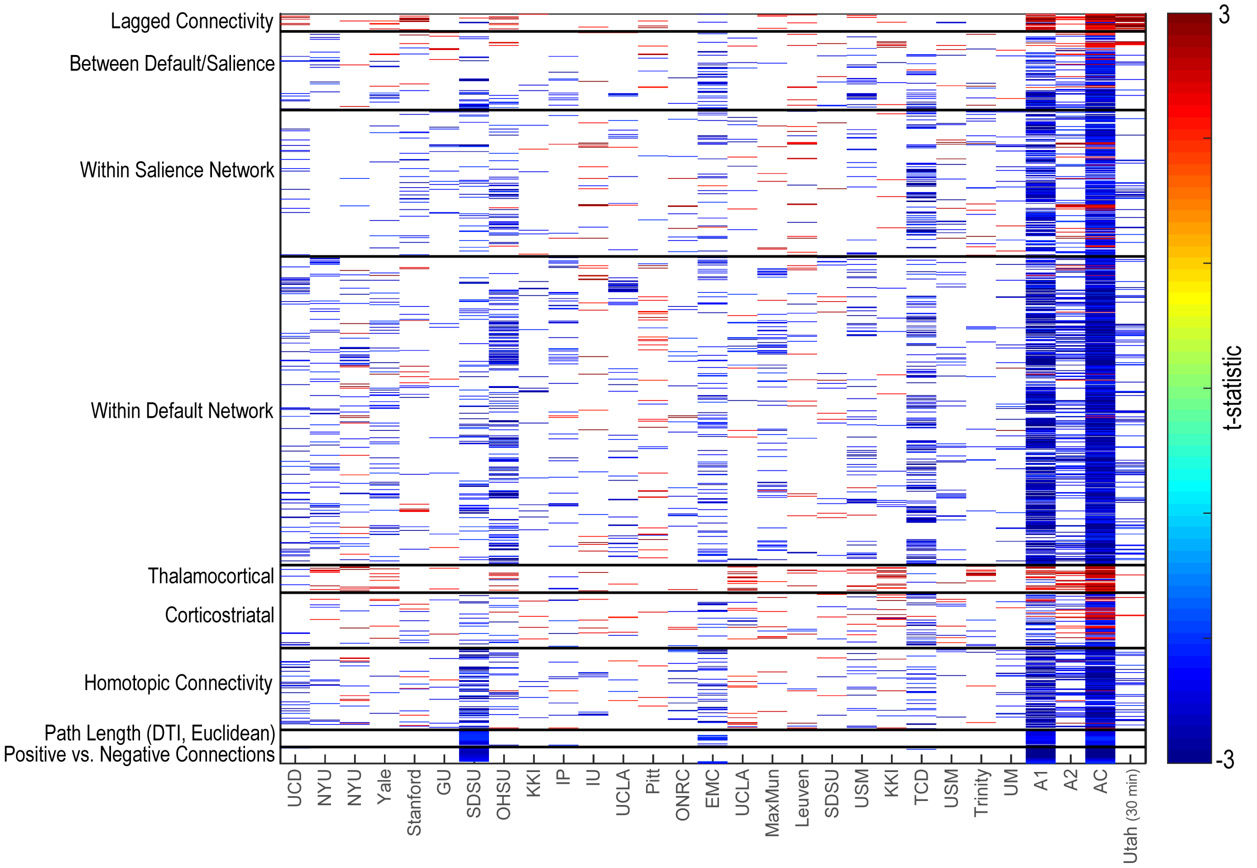

Supplementary Figure 11. Between-group comparison of resting-state functional connectivity methods sorted by eye status (open/closed). Distribution of between-group resting-state findings for select methods are presented (*p* < .05, uncorrected) for each research site, ABIDE I, ABIDE II, a combined ABIDE dataset, and a high temporal resolution replication sample (Utah cohort). Cooler colors represent autism < controls.

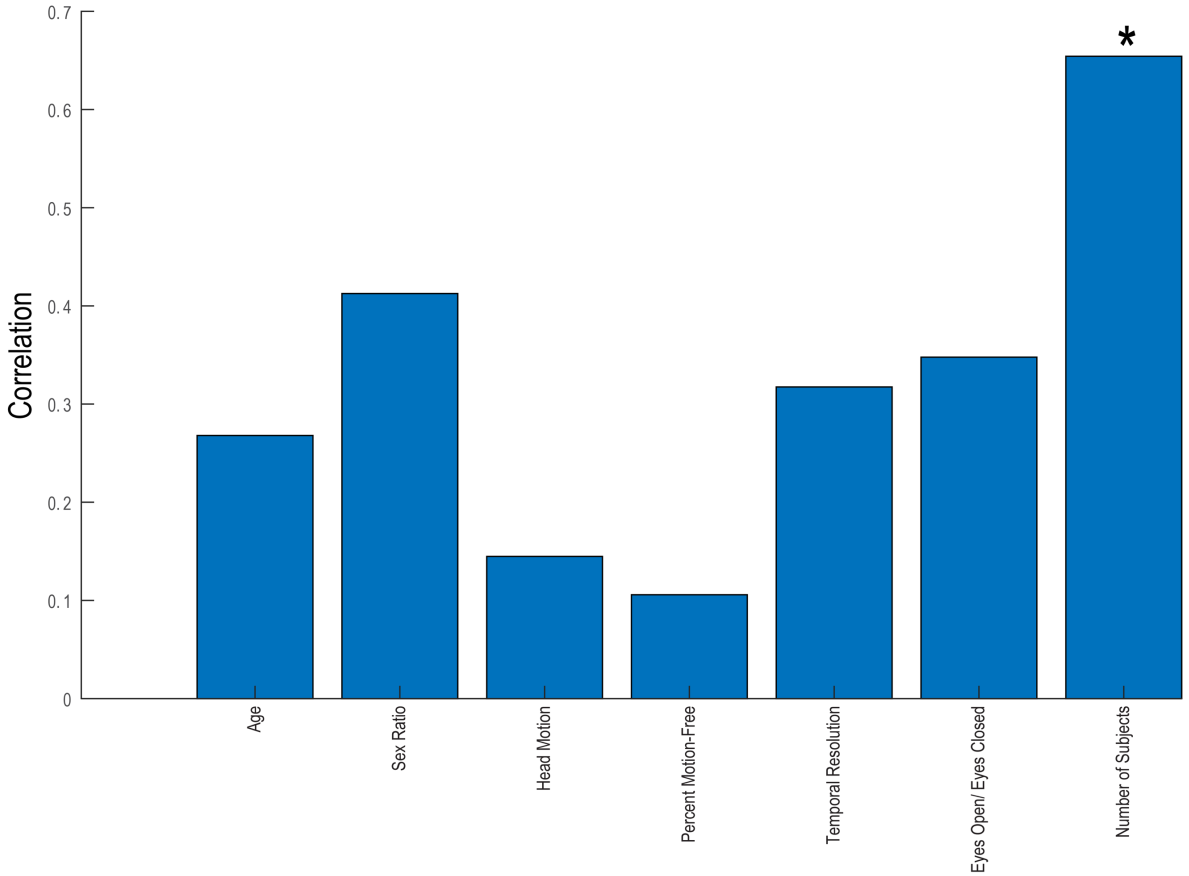

Supplementary Figure 12. Correlation between site specific factors and combined ABIDE results. The sample size of each site was the sole significant (*) factor that predicted the similarity of between-group comparison of resting-state functional connectivity methods from each site to the findings for the combined ABIDE sample.
